# Treating myocardial infarction via a nano-ultrasonic contrast agent-mediated high-efficiency drug delivery system targeting macrophages

**DOI:** 10.1126/sciadv.adp7126

**Published:** 2025-01-03

**Authors:** Zhen Ma, Ming Li, Rui Guo, Yu Tian, Yongbin Zheng, Bingxin Huang, Yi You, Qing Xu, Ming Cui, Li Shen, Feng Lan, Hang Yang, Rucong Liu, Tao Yang, Feng Wan, Qihua He, Xiao Huo, Youkun Bi, Yingying Zhang, Yunpeng Ling

**Affiliations:** ^1^ Department of Cardiac Surgery, Peking University Third Hospital, Beijing 100191, China.; ^2^ Joint International Research Center of Translational and Clinical Research, Beijing 100142, China.; ^3^ State Key Laboratory of Natural and Biomimetic Drugs, Beijing Key Laboratory of Molecular Pharmaceutics and Drug Delivery System, and School of Pharmaceutical Sciences, Peking University, Beijing 100191, China.; ^4^ Ultrasound Medical Center, Lanzhou University Second Hospital, Gansu Province Clinical Research Center for Ultrasonography, Gansu Province Medical Engineering Research Center for Intelligence Ultrasound, Lanzhou 730000, China.; ^5^ Peking University Shenzhen Graduate School, Shenzhen 518055, China.; ^6^ Department of Cardiology, Peking University Third Hospital, Beijing 100191, China.; ^7^ Department of Cell Biology, School of Basic Medical Sciences, Peking University Health Science Center, Beijing 100191, China.; ^8^ State Key Laboratory of Cardiovascular Disease, Fuwai Hospital, National Center for Cardiovascular Diseases, Chinese Academy of Medical Sciences and Peking Union Medical College, Beijing 100037, China.; ^9^ Department of Biomedical Informatics, State Key Laboratory of Vascular Homeostasis and Remodeling, School of Basic Medical Sciences, Peking University, Beijing 100191, China.; ^10^ Institute for Carbon Neutrality, Beijing Advanced Innovation Center for Materials Genome Engineering, University of Science and Technology Beijing, Beijing 100083, China.; ^11^ Department of Cardiac and Vascular Surgery, The First Affiliated Hospital of Kunming Medical University, Kunming 650032, China.; ^12^ Center of Basic Medical Research, Institute of Medical Innovation and Research, Peking University Third Hospital, Beijing 100191, China.; ^13^ Institute of Biophysics Chinese Academy of Sciences, Beijing 100101, China.; ^14^ School of Medical Imaging, Xuzhou Medical University, Xuzhou 221006, China.

## Abstract

Following myocardial infarction (MI), the accumulation of CD86-positive macrophages in the ischemic injury zone leads to secondary myocardial damage. Precise pharmacological intervention targeting this process remains challenging. This study engineered a nanotherapeutic delivery system with CD86-positive macrophage-specific targeting and ultrasound-responsive release capabilities. A folic acid (FA)–modified ultrasound-responsive gene/drug delivery system, assembled from DOTAP, DSPE-PEG2000-FA, cholesterol, and perfluorohexane (PFH)—termed FA-PNBs—was developed to codeliver small interfering RNA of STAT1 (siSTAT1) and the small-molecule nitro-oleic acid (OA-NO_2_) into CD86-positive macrophages. Upon irradiation with low-intensity focused ultrasound, FA-PNBs release siSTAT1 and OA-NO_2_ at the ischemic injury zone. The results demonstrated the system’s precise targeting and efficient delivery capabilities. The combined modulation of OA-NO_2_ and siSTAT1 optimizes the immune microenvironment in the infarcted region, alleviates ventricular remodeling, preserves cardiac function, and holds promise for clinical intervention strategies after MI.

## Teaser

FA-PNBs precisely target macrophages post-MI, codelivering OA-NO_2_ and siSTAT1 to optimize the immune microenvironment.

## INTRODUCTION

Ischemic heart disease remains a leading cause of mortality and disability worldwide, affecting approximately 126 million people globally (*1*, *2*). Myocardial infarction (MI) is the most severe form of ischemic heart disease (*3*). Now, adverse ventricular remodeling and heart failure following MI continue to be the primary contributors to patient mortality (*4*). Addressing these issues is a key focus of ischemic heart disease treatment, and mitigating post-MI adverse ventricular remodeling in patients with MI is a critical treatment objective.

Dysregulation of immune modulation plays a crucial role in ventricular remodeling after MI. After MI, monocytes and macrophages with a pro-inflammatory phenotype infiltrate the infarcted pro-inflammatory area to induce an inflammatory response and clear necrotic debris (*5*). This infiltration typically peaks approximately 3 days after MI (*6*). In the context of prolonged and severe inflammatory responses, adverse remodeling occurs, leading to heart failure. Hence, anti-inflammatory therapies are considered effective treatments for cardiac damage after MI.

M1 macrophages are classically pro-inflammatory cells that typically express CD86. Studies have shown that inhibiting CD86-positive macrophages may contribute to the treatment of MI (*7*). Proinflammatory CD86-positive macrophages can inhibit angiogenesis and exacerbate post-MI cardiac dysfunction (*8*). In contrast, Cx3cr1-positive macrophages are thought to contribute to recovery from MI (*9*). Thus, timely and targeted regulation of macrophage polarization is a potential therapeutic strategy for MI treatment.

Endogenous 9- and 10-nitro-octadec-9(E)-enoic acid [nitro-oleic acid (OA-NO_2_)] is a novel anti-inflammatory small-molecule compound that is being studied in the context of several diseases. Preclinical results indicate that OA-NO_2_ can facilitate repair in multiple systems of the body and reduce inflammation in the kidneys, cardiovascular system, and central nervous system; moreover, its safety and efficacy have been widely demonstrated, and it is now entering phase 2 clinical trials for treating glomerulosclerosis and pulmonary arterial hypertension (*10*). OA-NO_2_ functions by activating peroxisome proliferator–activated receptor (PPAR-γ) and inhibiting nuclear factor κB (NF-κB), which makes it capable of regulating anti-inflammatory macrophage formation (*11*–*13*). Signal transducers and activators of transcription 1 (STAT1) is a key molecule in regulating macrophage polarization and plays a notable role in preventing adverse remodeling after MI (*14*, *15*). Usually, STAT1 transcription factors promote pro-inflammatory macrophages (*16*). Given that NF-κB and STAT1 are key transcription factors that drive the formation of pro-inflammatory macrophages, while PPAR-γ plays a central role in promoting anti-inflammatory macrophages, this study sought to synergistically regulate macrophage polarization via OA-NO_2_ and siSTAT1, with the objective of converting pro-inflammatory macrophages into anti-inflammatory macrophages.

Therefore, inhibiting STAT1 to suppress pro-inflammatory (CD86-positive) macrophage formation and function, in combination with the OA-NO_2_–mediated promotion of Cx3cr1-positive macrophage polarization, could be an effective method for regulating the immune microenvironment in the infarct area. However, current targeted therapies for macrophages face limitations in terms of both organ targeting and drug-carrying efficiency. There is an urgent need for an efficient and precise multiplex drug delivery system that targets cardiac macrophages.

The folate receptor (FR), a cell surface glycoprotein with high affinity for folic acid (FA), facilitates the active uptake of FA. In contrast to the extremely low level of expression in most tissues, FRs are overexpressed in monocytes, especially activated CD86-positive macrophages. Therefore, FA, an excellent modifier that targets CD86-positive macrophages, began to be applied in targeted therapy (*17*, *18*).

Nanomedicine has emerged as a pivotal tool for targeted drug delivery to specific sites of interest. Now, the array of nanoparticles used in drug delivery encompasses thermo-responsive fluorescent nanoparticles (*19*), fluorescent pH-responsive nanoparticles (*20*), and others. The widespread adoption of ultrasound (US) technology in the cardiovascular arena, attributed to its precision in localization and minimal invasiveness, underscores its potential utility. However, the exploration of US-responsive nanomaterials for the targeted delivery of therapeutics to areas affected by MI is notably limited. This observation underscores a pressing need for further research into the deployment of US-responsive nanoparticles for precision drug delivery to the MI zone, representing a promising avenue for advancing cardiovascular disease management.

US-mediated drug release from nanoparticles has shown promise in preventing premature drug release in nontargeted tissues and facilitating drug entry into cells under US (*21*). Low-intensity focused ultrasound (LIFU) combined with nanomaterials can exert biological effects through mechanisms such as cavitation, mechanical, thermal, and acoustic pore effects and has been widely used in drug delivery (*22*). Under the excitation of the mechanical and thermal effects of LIFU, liquid-gas phase transition materials can undergo acoustic droplet vaporization (ADV) to generate gas micro/NBs (nanobubbles), thereby increasing the US imaging ability (*23*). One such phase change material is perfluorohexane (PFH), which is a liquid at room temperature and exhibits good biocompatibility. PFH can be effectively encapsulated into liposome nanoparticles, and under the excitation of LIFU, it undergoes a phase transition to a gas state, forming micro/NBs that enhance US imaging. Furthermore, the continuous liquid-gas bursting of PFH during continuous excitation, combined with the unique sonoporation effect and cavitation of US, can promote targeted drug/gene delivery and achieve individualized treatment (*24*).

The aim of this study was to engineer a nanotherapeutic delivery system with CD86-positive macrophage-specific targeting and US-triggered release capabilities by leveraging nano-US contrast agents and exploiting the unique acoustic cavitation effect of US. We developed a highly precise and efficient US-responsive nanomedicine delivery system that targets CD86-positive macrophages after MI, enabling the simultaneous delivery of nucleic acids and compound drugs. Targeted small interfering RNA (siRNA) release and OA-NO_2_ combined therapy using FA-PNBs drug carrier system in MI area notably enhance the immune microenvironment after MI and show promising potential for clinical translation.

## RESULTS

### The synthesis process of OA-si@FA-PNBs and a schematic illustration of their ability to improve MI in mice via US assistance

**Fig. 1.**
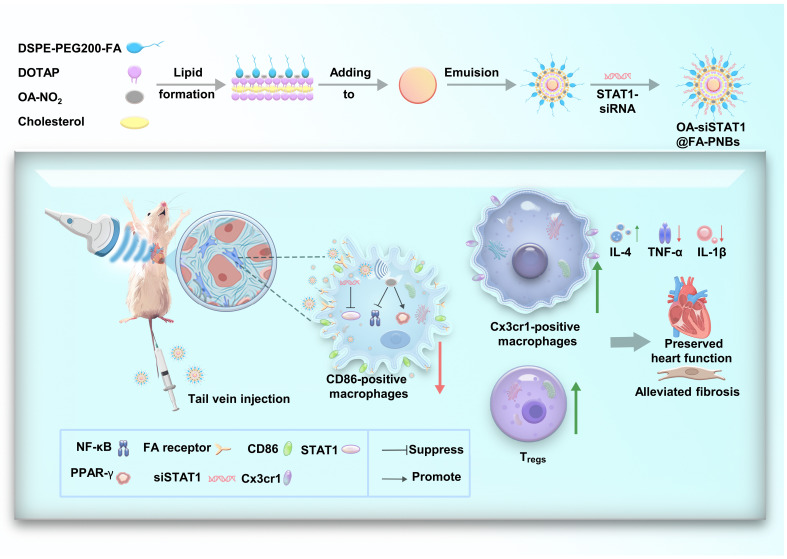
Graphical abstract of US-responsive nanoparticles. Synthesis process of OA-si@FA-PNBs and a schematic illustration of their effects on improving MI in mice under US assistance.

The gene/drug delivery system was termed the FA–perfluorohexane nanobubbles (FA-PNBs) system. In this study, FA-PNBs carrying both nitro-fatty acids (OA-NO_2_) and STAT1-interfering RNA (siSTAT1) were investigated as innovative immunotherapeutic agents. LIFU irradiation induced a phase transition in PFH from liquid to gas, which enhanced the US imaging capabilities and precise delivery of the compound and genetic therapeutics. These NBs were synthesized via a classical thin-film hydration method augmented by sonication to obtain a core-shell architecture. The hydrophobic OA-NO_2_ was integrated into the lipid bilayer to form the shell, and PFH was encapsulated within the core. si@FA-PNBs indicate that the drug delivery system carried siSTAT1, OA@FA-PNBs indicate that the drug delivery system carried OA-NO_2_, and OA-si@FA-PNBs indicate that the drug delivery system carried OA-NO_2_ and siSTAT1 (Fig. 1).

### Characterization and performance of FA-PNBs loaded with siRNA and nitro-fatty acids: Stability, imaging, and transfection efficiency

**Fig. 2.**
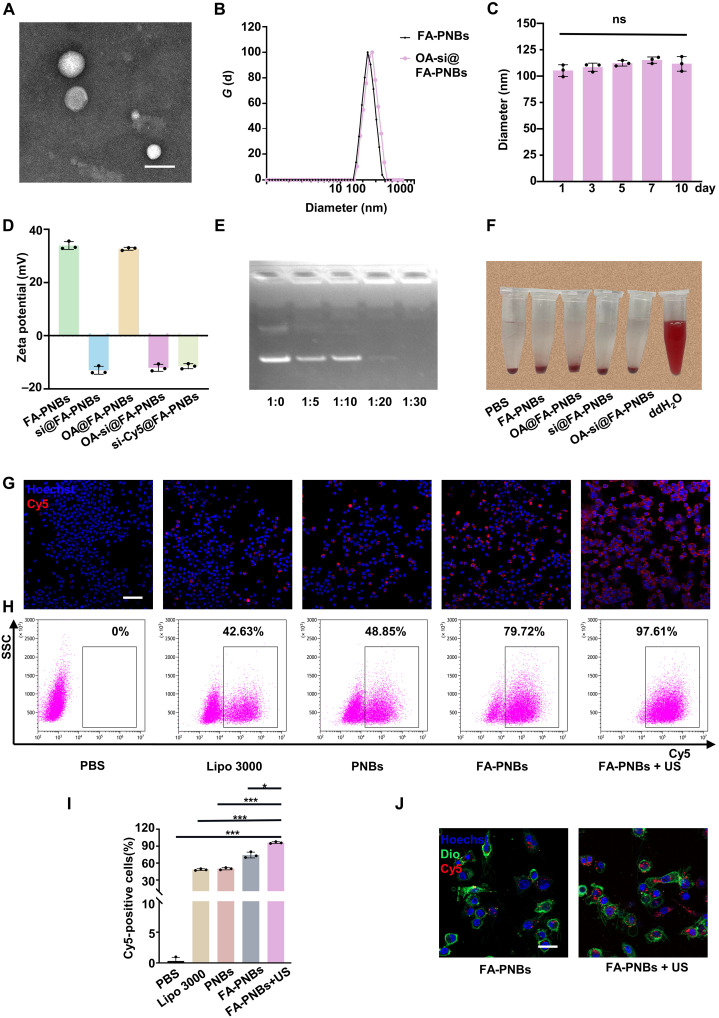
Characterization and performance of FA-PNBs loaded with siRNA and nitro-fatty acids: Stability, imaging, and transfection efficiency. (**A**) TEM image. Scale bar, 100 nm. (**B**) Size distribution analysis of FA-PNBs and OA-si@FA-PNBs. (**C**) Stability of the OA-si@FA-PNBs. (**D**) Surface zeta potential of the FA-PNBs, si@FA-PNBs, OA@FA-PNBs, OA-si@FA-PNBs, and si-Cy5@FA-PNBs. (**E**) Agarose gel electrophoresis of FA-PNBs loaded with siSTAT1. (**F**) Hemolysis experiment. Equal volumes of PBS, FA-PNBs, si@FA-PNBs, OA@FA-PNBs, OA-si@FA-PNBs, and ddH_2_O were added to the blood cells of the mice. (**G**) Confocal images of Cy5-tagged siSTAT1 under different transfection conditions. Red indicates Cy5-conjugated siRNA (siSTAT1-Cy5). Scale bar, 50 μm. (**H**) Flow cytometry analysis of Cy5-tagged siSTAT1 under various conditions. (**I**) Statistical analysis of the flow cytometry results. (**J**) Confocal imaging of DiO-labeled cell membranes (green) and Cy5-conjugated OA-si@FA-PNBs (red) under different conditions. Scale bar, 25 μm. siSTAT1, siRNA targeting STAT1. The data are expressed as the means ± SDs. *n* = 3 per group. *P < 0.05 and ***P < 0.001. ns, not significant.

The size and distribution of the OA-si@FA-PNBs, as observed through transmission electron microscopy (TEM), were uniform (Fig. 2A). These spherical NBs were approximately 100 nm in size, which was consistent with the dimensions assessed via a Malvern Nano analyzer (Fig. 2B). Stability tests over 10 days revealed no significant alterations in size (*P* > 0.05), underscoring the robustness of the NBs (Fig. 2C). The zeta potentials of the empty nanocarrier control FA-PNBs (NC@FA-PNBs), FA-PNBs loaded with siSTAT1 (si@FA-PNBs), FA-PNBs loaded with OA-NO_2_ (OA@FA-PNBs), OA-si@FA-PNBs, and si-Cy5@FA-PNBs were 33.9 ± 1.2, −12.97 ± 1.21, 32.72 ± 0.44, −12.03 ± 1.08 and − 11.43 ± 0.78 mV, respectively (Fig. 2D). Furthermore, the nucleic acid–carrying capacity of the FA-PNBs was validated via agarose gel electrophoresis. The absence of siRNA bands at a siSTAT1 to FA-PNB mass ratio of 1:20 indicated efficient nucleic acid encapsulation (Fig. 2E). The hemolysis assay results corroborated the biocompatibility of the NBs, with no erythrocyte damage observed under the experimental conditions, in contrast to the positive control (Fig. 2F). In addition, we assessed the stability of FA-PNBs in serum. The results revealed that siSTAT1 carried by FA-PNBs remained stable after 12 hours of incubation, whereas free siSTAT1 was completely degraded during this period (fig. S1, A and B). PFH has emerged as an optimal phase-transition material that is stable as a liquid at room temperature but can be converted to gas upon activation by US, temperature, or laser stimuli (fig. S1C). Within the FA-PNBs, PFH facilitated ADV post-LIFU, increasing the US imaging efficacy. Pre-LIFU scans revealed minimal echoes, which significantly increased upon adjusting the LIFU parameters to 3 W/cm^2^ for 3 min (fig. S1, D and E). Therefore, from an imaging perspective, the optimal LIFU excitation intensity for NBs is 3 W/cm^2^ for 3 min. In addition, we evaluated the safety of ultrasonic intensity and duration on mouse cardiomyocytes (HL-1) and RAW264.7 cells and found that ultrasonic treatment was nontoxic to cells when the sound intensity was less than 5 W/cm^2^, and the duration was less than 5 min (fig. S1, F and G). We used Cy5-conjugated siSTAT1 and confocal microscopy and flow cytometry to evaluate the transfection efficiency across various CD86-positive macrophage treatments. In the phosphate-buffered saline (PBS) group, macrophage culture medium supplemented with PBS without siRNA was used as the control group. For the cationic liposome (Lipo) group, siRNA was added to the macrophage culture medium with Lipo 3000. In the PNBs group, macrophage culture medium was supplemented with PNBs without FA modification. In the FA-PNBs group, FA-PNBs with FA modification were added to the macrophage culture medium, and the siRNA was adsorbed in the FA-PNBs. In the FA-PNBs + US group, FA-PNBs were added to the macrophage culture medium, and siRNA was adsorbed in the FA-PNBs. In addition, the cells were subjected to US irradiation. Immunofluorescence revealed the most intense fluorescence in the FA-PNBs + US group, followed by the FA-PNBs group without US, which had markedly brighter fluorescence than the non-FA–modified PNB and conventional lipofection groups did (Fig. 2G). These results suggested that FA modification and US irradiation promoted Cy5-siSTAT1 transfection into cells. Flow cytometry confirmed these findings (Fig. 2H), with quantitative analysis revealing that the transfection efficiency in the FA-PNBs + US group was significantly greater than that in the other groups (Fig. 2I). This demonstrated that combining FA-PNBs with US achieved high-efficiency transfection. The flow cytometry gating strategy is detailed in fig. S1H. Further confocal microscopy analysis revealed greater intracellular nanoparticle uptake in the FA-PNBs with US group than in the FA-PNBs without US group (Fig. 2J). The OA-NO_2_ content was determined via atomic absorption spectroscopy (*R*^2^ = 0.9911), and the OA-NO_2_ encapsulation efficiency and loading capacity were calculated to be 81.33 ± 1.57% and 16.9 ± 0.27%, respectively (fig. S1I).

### FA-modified PNBs (OA-si-Cy5@FA-PNBs) can be notably enriched in the myocardial infarction area and US can extend the enrichment time of PNBs

**Fig. 3.**
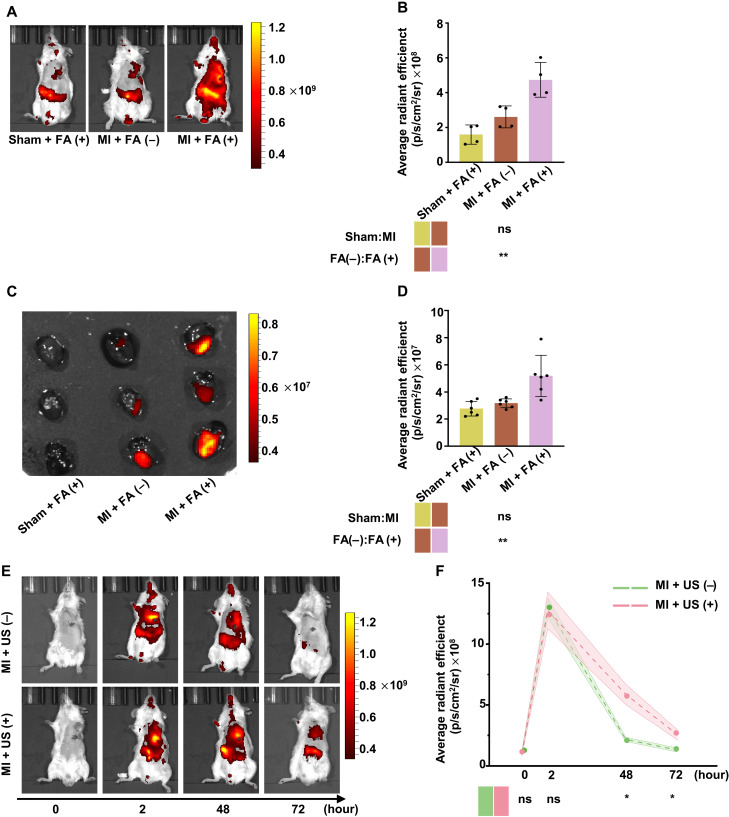
FA-modified PNBs (OA-si-Cy5@FA-PNBs) were notably enriched in the MI area. US can extend the enrichment time of these materials. (**A**) Live optical imaging of sham and MI model mice 2 hours after FA-PNB or PNB injection. Sham + FA (+) indicates sham mice that received tail vein injections of PNBs with FA modification. MI + FA (−) indicates MI mice that received tail vein injections of PNBs without FA modification. MI + FA (+) indicates MI mice that received tail vein injections of FA-PNBs. (**B**) Quantification of fluorescence intensity in the precordial area of the heart. *n* = 4 per group. (**C**) Ex vivo optical imaging of infarcted hearts from sham and MI model mice 2 hours postinjection. (**D**) Statistical analysis of the average fluorescence intensity in ex vivo hearts. *n* = 6 per group. (**E**) Live optical imaging after intravenous injection of FA-PNBs with or without external US treatment at 0, 2, 48, and 72 hours. MI + US (+) indicates MI mice that were irradiated with US for 3 min after tail vein injection. (**F**) Quantitative analysis of fluorescence intensity in the precordial area of the heart. *n* = 3 per group. FRβ, FA receptor β; FA, folic acid. The data are expressed as the means ± SDs. *P < 0.05, **P < 0.01, and ***P < 0.001.

In vivo targeting of siSTAT1-Cy5 packaged in FA-PNBs was monitored via optical imaging, with the heart Cy5 intensity peaking 2 hours after intravenous injection (fig. S2, A and B). Therefore, in our animal experiments, the mice received US irradiation 2 hours after tail vein injection. To assess whether FA-PNBs could be used for targeted drug delivery to infarcted hearts, we evaluated the organ accumulation of FA-PNBs after MI. We found that MI mice injected with FA-modified NBs [MI + FA (+)] had significantly greater fluorescence intensity in the heart region than sham mice [sham + FA (+)]. In addition, we found that in MI model mice injected with FA-modified PNBs [MI + FA (+)], NBs accumulated more in the MI region than did those without FA-modified PNBs [MI + FA (−)] (Fig. 3, A and B). To further clarify the cardiac targeting of FA-PNBs, we dissected the above three groups of mice, removed important organs (heart, liver, lung, spleen, and kidney), and conducted ex vivo optical imaging. A comparison of Cy5 accumulation in major organs revealed increased levels of Cy5 in the heart (Fig. 3, C and D) and liver (fig. S2, C and D). The results were consistent with those of ex vivo optical imaging. Together, these results suggest that FA-PNBs may be potential carriers for targeted drug delivery to the infarcted heart after MI. To explore the residence time of FA-PNBs in the heart region, MI mice were subjected to in vivo imaging detection at 0, 2, 48, and 72 hours after tail vein injection. We found that in mice that received FA-PNBs only [MI + US (−)], the maximum accumulation time of FA-NBs in the cardiac region was 2 hours after injection, and the FA-NB accumulation time almost gradually decreased and almost disappeared after 48 and 72 hours. However, in combination with US irradiation (3 W/cm^2^, 3 min) [MI + US (+)], many FA-PNBs remained in the heart region at 48 and 72 hours after injection, which was significantly greater than that in the MI + US (−) mice (Fig. 3, E and F). These findings suggest that US irradiation can increase the retention time of FA-PNBs in the heart region.

### Precision codelivery of siSTAT1 and OA-NO_2_ to CD86-positive macrophages in MI via FA-modified nanoparticles

**Fig. 4.**
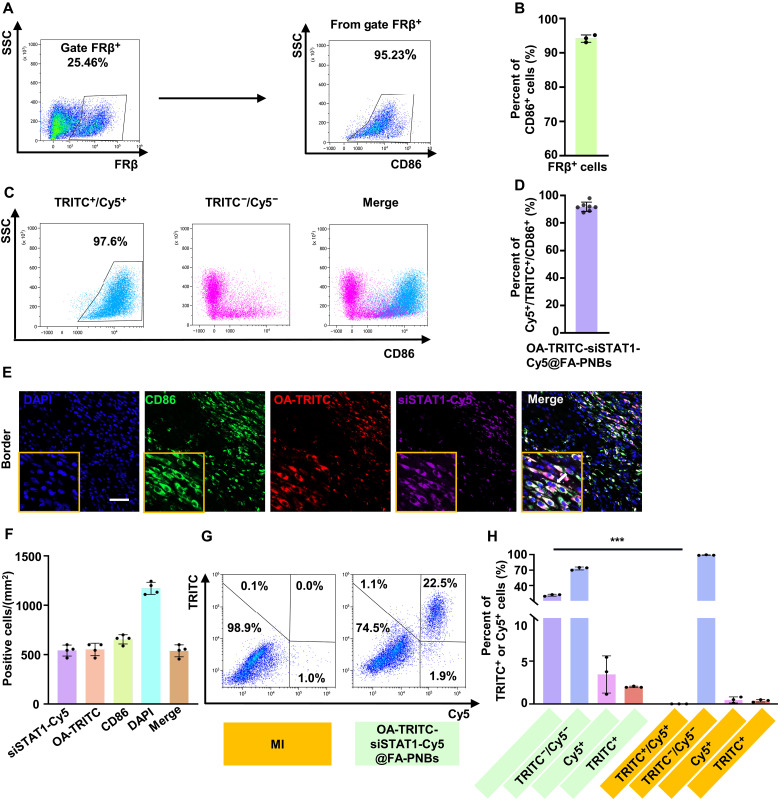
Precision codelivery of siSTAT1 and OA-NO_2_ to CD86-positive macrophages in MI using FA-modified nanoparticles. (**A**) Flow cytometry analysis of FRβ-positive and CD86-positive cells in the anterior wall of the left ventricle of MI mice on day 3. (**B**) Proportion of CD86-positive cells among FRβ-positive cells was 95.23%, as determined by flow cytometry. *n* = 3 per group. (**C**) Flow cytometry analysis of CD86-positive cells among TRITC^+^/Cy5^+^ cells in the MI area. siSTAT1 was labeled with Cy5, OA-NO_2_ was labeled with TRITC, and OA-TRITC-siSTAT1-Cy5@FA-PNBs were administered via tail vein injection 3 days post-MI. (**D**) Quantification of the flow cytometry data revealed that 97.6% of the TRITC^+^/Cy5^+^ cells were CD86 positive. *n* = 7 per group. (**E**) Confocal microscopy detection of Cy5, TRITC, and CD86 triple-positive cells in the ischemic region. The arrows indicate triple-positive CD86, OA-TRITC, and siSTAT1-Cy5 cells. Scale bar, 50 μm. (**F**) Quantitative analysis of Cy5-positive, TRITC-positive, CD86-positive, and triple-positive cells in the ischemic region. *n* = 4 per group. (**G**) Flow cytometry assessment of Cy5 and TRITC double-positive, Cy5-only, TRITC-only, and double-negative cells in the ischemic myocardial injury region. (**H**) Quantification of these populations from the flow cytometry results. The data are expressed as the means ± SDs. *n* = 3 per group. TRITC, tetramethylrhodamine isothiocyanate. ***P < 0.001.

In addition, we conducted a more detailed analysis of the dissected hearts to investigate the specific cell populations targeted by FA-PNBs in the cardiac tissue. We found that FRβ expression was greater in CD86-positive macrophages than in CD86-negative macrophages, NIH3T3 cells, and HL-1 cells (fig. S3, A and B). We further investigated the relationship between FRβ- and CD86-positive cells in the infarcted area and found that on day 3 post-MI, 25.46% of the cells expressed FRβ, and 95.23% of the FRβ-positive cells were CD86-positive macrophages (Fig. 4, A and B). The results of confocal microscopy were consistent with these findings, as CD86 (green) and FRβ (red) were colocalized (fig. S3C). The flow cytometry gating strategy is detailed in fig. S3D. Only 3.32% of the FRβ-positive cells were CD86^−^/CD31^+^ endothelial cells, and 13.51% were CD86^−^/vimentin^+^ fibroblasts (fig. S3D).

We subsequently injected OA-si-Cy5@FA-PNBs into MI mice and sorted the Cy5-positive and Cy5-negative cells. Through single-cell RNA sequencing (scRNA-seq) analysis, we identified 21 distinct clusters. The primary cell subgroups included macrophages and other immune cells, such as dendritic cells, eosinophils, plasma cells, mast cells, B cells, and T cells, alongside minor populations of other cell types, including smooth muscle cells, cardiomyocytes, epithelial cells, and vascular endothelial cells (fig. S3E). scRNA-seq analysis revealed that Cy5-positive cells highly expressed the CD86 gene (73.3%), whereas Cy5-negative cells presented minimal to no CD86 expression (9%; fig. S3, F and G). Notably, Cy5-positive cells were predominantly macrophages (68.35%; fig. S3H). These findings suggest that following MI, the majority of cells targeted by PNBs in vivo are CD86-positive macrophages.

To further investigate whether FA-PNBs carrying both siSTAT1 and OA-NO_2_ can simultaneously target CD86-positive macrophages within the heart, we labeled siSTAT1 with Cy5 and OA-NO_2_ with tetramethyl rhodamine isothiocyanate (TRITC). Infrared spectroscopy confirmed the successful conjugation of TRITC to OA-NO_2_ (fig. S3, I and J). Three days after MI induction, we injected FA-PNBs carrying both siSTAT1-Cy5 and OA-TRITC via the tail vein. After an US-induced burst, the anterior wall of the left ventricle (LV) was excised, dissociated into a single-cell suspension, and analyzed via flow cytometry. The results revealed that 97.6% of the cells that were double positive for Cy5 and TRITC were CD86-positive macrophages (Fig. 4, C and D), with only approximately 1.16% being endothelial cells or 0.23% being fibroblasts (fig. S3K). Confocal microscopy revealed a strong correspondence between Cy5-positive and TRITC-positive cells and the CD86-positive cell population (Fig. 4, E and F). Notably, no Cy5 or TRITC signals were detected in untreated cardiac tissue within the infarcted area (fig. S3L). We also evaluated the proportions of Cy5 and TRITC double-positive, Cy5 and TRITC double-negative, CD86-positive, and TRITC-positive cells in the left ventricular anterior wall of MI mice treated with siSTAT1-Cy5-OA-TRITC@FA-PNBs. The results revealed that 22.5% of the cells coexpressed Cy5 and TRITC, indicating that our FA-PNBs system effectively delivered both siSTAT1 and OA-NO_2_ into the same cell. Only 3% of the cells expressed either Cy5 or TRITC alone (Fig. 4, G and H).

### Synergistic regulation of the macrophage phenotype by OA-NO_2_ and siSTAT1

**Fig. 5.**
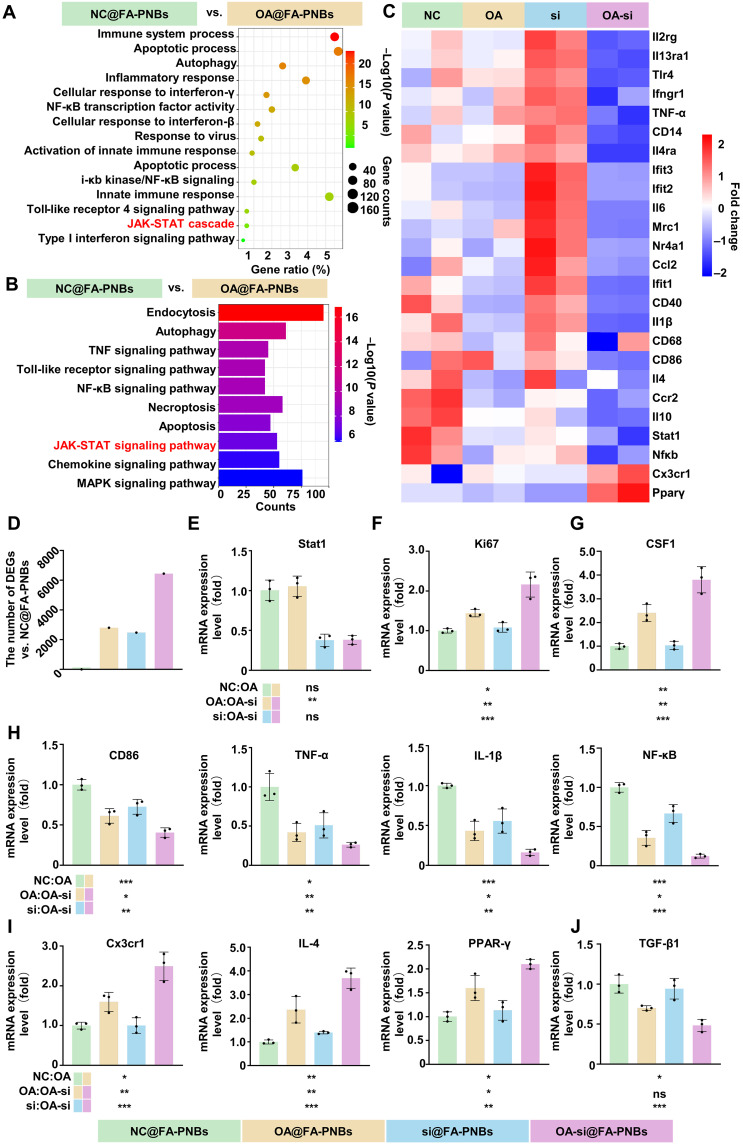
Transcriptome analysis of macrophages treated with different FA-PNBs. (**A**) Gene Ontology (GO) analysis highlighting the biological processes in CD86-positive macrophages treated with OA@FA-PNBs versus NC@FA-PNBs. (**B**) KEGG pathway analysis of the DEGs. (**C**) Heatmap of inflammation-related gene expression changes in the various groups. (**D**) Quantitative differential gene expression. (**E** to **J**) qRT-PCR quantification of macrophage-related gene expression levels. Green, NC@FA-PNBs group; yellow, OA@FA-PNBs group; blue, si@FA-PNBs group; purple, OA-si@FA-PNBs group. US, ultrasound; MI, myocardial infarction; FA-PNBs, folic acid-perfluorohexane nanobubble system; PNBs, perfluorohexane nanobubble system; siSTAT1, siRNA targeting STAT1; OA-NO_2_, nitric acid; FA, folic acid. The data are presented as the means ± SDs, *n* = 3 per group. *P < 0.05, **P < 0.01, ***P < 0.001, ns: not significant.

To explore the regulatory effects of OA-NO_2_ on cardiac macrophages, we conducted an unbiased transcriptomic analysis. Transcriptome sequencing of CD86-positive macrophages treated with OA-NO_2_ followed by analysis of enriched differentially expressed genes (DEGs) via Gene Ontology (GO) and Kyoto Encyclopedia of Genes and Genomes (KEGG) enrichment analyses suggested a pivotal role for the Janus kinase (JAK)–STAT pathway in OA-NO_2_–mediated modulation of the macrophage phenotype (Fig. 5, A and B). Given the critical role of STAT1 in the JAK-STAT pathway and its role as a key transcription factor that promotes CD86-positive macrophage formation, we further explored the regulatory effects of OA-NO_2_ on the macrophage phenotype via the use of siSTT1. Combined OA-NO_2_ and siSTAT1 treatment of CD86-positive macrophages resulted in a significant reduction in the expression of pro-inflammatory cytokine-associated genes [CD86, tumor necrosis factor–α (TNF-α), interleukin-1β (IL-1β), NF-κB, and CD40] and an increase in the expression of tissue-repairing associated genes (PPAR-γ, Cx3cr1, and IL-4) (Fig. 5C). In addition, a marked increase in differential gene expression was observed after treatment (Fig. 5D). Quantitative reverse transcription polymerase chain reaction (qRT-PCR) revealed a more pronounced decrease in STAT1 expression in the si@FA-PNBs group than in the NC@FA-PNBs group (Fig. 5E). In addition, this regimen significantly promoted the expression of genes associated with macrophage proliferation. The expression of Ki67 and CSF1 was significantly increased in the OA-si@FA-PNBs group (Fig. 5, F and G). Compared with that in NC@FA-PNBs, the expression of proinflammatory cytokine-associated genes (CD86, TNF-α, IL-1β, and NF-κB) was significantly down-regulated in OA-si@FA-PNBs (Fig. 5H), and the expression of tissue-repairing associated genes (PPAR-γ, Cx3cr1, and IL-4) was significantly up-regulated (Fig. 5I). While promoting Cx3cr1-positive polarization, OA-NO_2_ combined with siSTAT1 did not increase the secretion of transforming growth factor–β1 (TGF-β1), a critical fibrosis-inducing factor, which tends to induce malignant ventricular remodeling (Fig. 5J).

Cell viability assessments via Cell Counting Kit 8 (CCK-8) assays demonstrated a marginal decrease in RAW264.7 cell viability with increasing concentrations of the various NB formulations; however, an overall survival rate above 90% was maintained, even in cells treated with NBs at 200 μg/ml. Therefore, the concentration used in this experiment was 200 μg/ml (fig. S4A). To further clarify the relationship between OA-NO_2_ and STAT1, we conducted additional experiments using macrophages with STAT1 overexpression (STAT1-OE). We observed that in the STAT1-OE macrophage line, the ability of OA-NO_2_ to inhibit CD86-positive macrophages was significantly diminished (fig. S4, B and C). These findings suggest that STAT1 inhibits the effects of OA-NO_2_, which is why we selected the combination of OA-NO_2_ with siSTAT1 to more effectively regulate macrophage polarization.

In addition, we determined the optimal concentration of OA-NO_2_ for activating PPAR-γ through qPCR. The results indicated that the expression of PPAR-γ was highest at a concentration of 1 μM OA-NO_2_ (fig. S4D). In addition, we explored the toxicity of OA-NO_2_ to RAW264.7 macrophages via the CCK-8 method. OA-NO_2_ had no obvious toxic effects on cells at concentrations lower than 6 μM (fig. S4E).

### OA-si@FA-PNBs modulate the macrophage phenotype and reduce inflammation in vitro

**Fig. 6.**
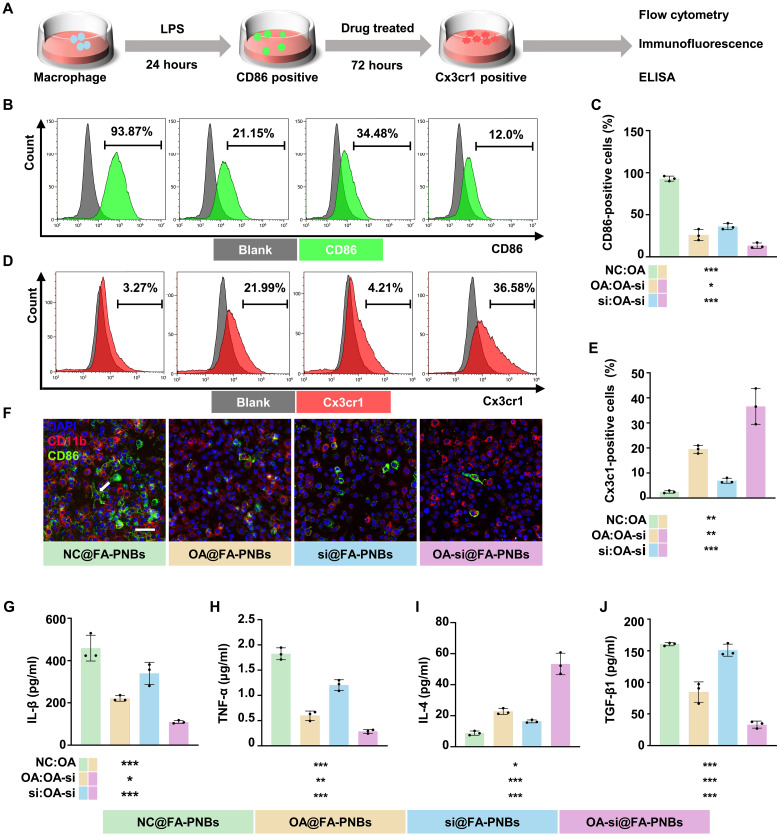
OA-si@FA-PNBs modulated the macrophage phenotype and reduced inflammation in vitro. Twenty-four hours after LPS induction, CD86-positive macrophages were treated with NC@FA-PNBs, OA@FA-PNBs, si@FA-PNBs, or OA-si@FA-PNBs and subjected to ultrasonic stimulation for 3 min. The results were analyzed 3 days after treatment. (**A**) Schematic showing the procedures used for macrophage polarization and phenotype detection. (**B**) Proportion of CD86-positive cells was detected by flow cytometry. (**C**) Statistical map showing the proportion of cells positive for CD86. (**D**) Proportion of Cx3cr1-positive cells was detected by flow cytometry. (**E**) Statistical map showing the proportion of Cx3cr1-positive cells. (**F**) Scanning confocal microscopy detection of CD86 (green), CD11b (red) and DAPI (blue) expression. Scale bar, 50 μm. The arrows indicate cells that are double positive for both CD86 and CD11b. (**G** and **H**) CD86-positive macrophage markers IL-1β and TNF-α were detected via ELISA. (**I** and **J**) ELISA detection of IL-4 and TGF-β1. Green, NC@FA-PNBs group; yellow:,OA@FA-PNBs group; blue, si@FA-PNBs group; purple, OA-si@FA-PNBs group. The data are expressed as the means ± SDs. *n* = 3 per group. *P < 0.05, **P < 0.01, and ***P < 0.001.

To explore the ability of FA-PNBs to regulate the macrophage phenotype, normal RAW264.7 macrophages were induced to differentiate into the phenotype by lipopolysaccharide (LPS) stimulation and then treated with NC@FA-PNBs, OA@FA-PNBs, si@FA-PNBs, or OA-si@FA-PNBs and subjected to ultrasonic stimulation for 3 min. Three days later, flow cytometry, immunofluorescence and enzyme-linked immunosorbent assay (ELISA) were performed to detect the polarization phenotype of the macrophages (Fig. 6A). Compared with those in the NC@FA-PNBs control group, a significant increase in the number of Cx3cr1-positive cells and a decrease in the number of CD86-positive cells were observed in the OA-si@FA-PNBs group. A notable reduction in CD86-positive cells and a slight increase in Cx3cr1-positive cells were also observed in the OA@FA-PNBs group. A significant reduction in the number of CD86-positive cells was observed in the si@FA-PNBs group (Fig. 6, B to E). The gating strategy for flow cytometry is detailed in the Supplementary Materials (fig. S5, A and B).

In addition, the numbers of cells double-positive for CD86 (green) and CD11b (red) were lower in the OA-si@FA-PNBs group than in the in the NC@FA-PNBs, OA@FA-PNBs, and si@FA-PNBs groups (Fig. 6F). ELISA analysis confirmed a significant decrease in IL-1β and TNF-α secretion (Fig. 6, G and H) and an increase in IL-4 secretion in the OA-si@FA-PNBs group, along with a decrease in the secretion of TGF-β1, a key fibrosis-inducing factor (Fig. 6, I and J).

### Nanocomplexes modulate the immune microenvironment of heart tissue in MI model mice

**Fig. 7.**
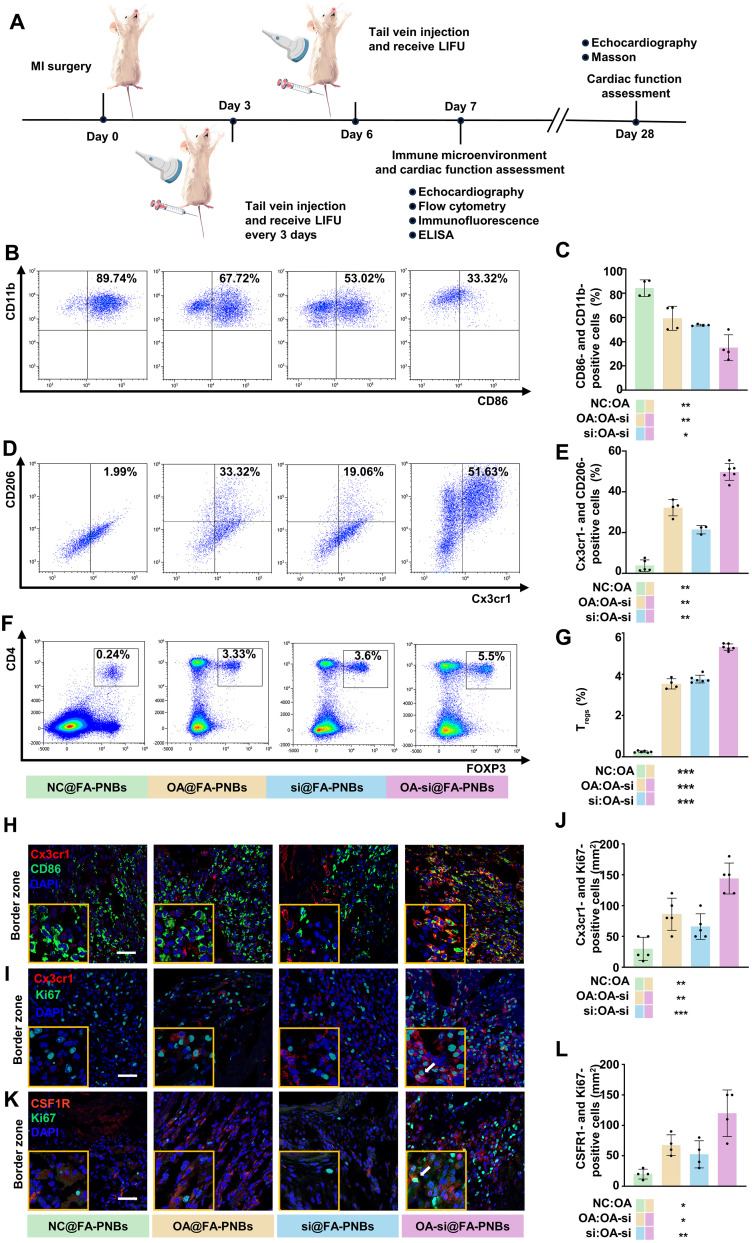
OA-si@FA-PNBs improve the post-MI immune microenvironment in mice. (**A**) Schematic showing the procedures of evaluating immune microenvironment and cardiac function in MI mice. (**B**) Flow cytometry analysis and the quantification (**C**) of the proportion of CD86-positive macrophages in the ischemic myocardial injury region of MI mice under different treatments. *n* = 4 per group. (**D**) Flow cytometry analysis and the quantification (**E**) of the proportion of Cx3cr1-positive macrophages in the ischemic region of MI mice under different treatments. (NC@FA-PNBs group, *n* = 5; OA@FA-PNBs groups, *n* = 4; si@FA-PNBs group, *n* = 3; OA-si@FA-PNBs group, *n* = 6). (**F**) Flow cytometry analysis and the Quantification (**G**) of the proportion of Tregs (CD4^+^/FOXP3^+^ cells) in the ischemic region of MI mice under different treatments. (NC@FA-PNBs group, *n* = 6; OA@FA-PNBs groups, *n* = 4; si@FA-PNBs group, *n* = 6; OA-si@FA-PNBs group, *n* = 6). (**H**) Immunostaining of MI mice on day 7 post-MI for the CD86 (green) and Cx3cr1 (red) in the ischemic border zones. (**I**) Immunostaining of MI mice on day 7 post-MI for the Cx3cr1 (red) and Ki67 (green) the ischemic border zones. The arrows indicate cells that are double positive for both Cx3cr1 and Ki67. (**J**) Quantitative analysis of Cx3cr1-positive and Ki67-positive cells/mm^2^. *n* = 5 per group. (**K**) Immunostaining of MI mice on day 7 post-MI for the CSF1R (red) and Ki67 (green) in the ischemic border zones. The arrows indicate cells that are double positive for both CSF1R and Ki67. (**L**) Quantitative analysis of CSF1R-positive and Ki67-positive cells/ mm2. *n* = 4 per group. Scale bar, 50 μm. The data are expressed as the means ± SD. *P < 0.05, **P < 0.01, and ***P < 0.001.

To explore the regulatory effect of PNBs on the cardiac immune microenvironment in MI model mice, we administered NC@FA-PNBs, OA@FA-PNBs, si@FA-PNBs, or OA-si@FA-PNBs to MI model mice via tail vein injection 3 days after modeling. In addition, US was applied to the anterior zone of the heart. After 7 days of MI surgery, heart tissue was collected for flow cytometry, confocal microscopy, and ELISA experiments (Fig. 7A). We used CD11b, F4/80, and CD86 to characterize CD86-positive macrophages and CD11b, F4/80, and Cx3cr1 to characterize Cx3cr1-positive macrophages. The results of flow cytometry revealed that the proportion of CD86-positive cells was significantly reduced in the OA-si@FA-PNBs group, whereas the proportion of Cx3cr1-positive cells was significantly increased. The administration of OA@FA-PNBs increased the proportion of Cx3cr1-positive cells and decreased the proportion of CD86-positive cells to a certain extent. (Fig. 7, B to E). In addition, we observed an increase in the number of regulatory T cells (T_regs_) in the hearts of OA-si@FA-PNBs. T_regs_ are effective negative regulators of inflammation in various biological environments and inhibit the proinflammatory properties of macrophages, which is a key factor in improving the MI immune microenvironment. We used CD4 and Foxp3 to characterize T_regs_. The flow cytometry results revealed a significant increase in the proportion of T_regs_ in the OA-si@FA-PNBs group compared with that in the NC@FA-PNBs, OA@FA-PNBs, and si@FA-PNBs groups (Fig. 7, F and G). The gating strategy is detailed in fig. S6 (A to C). We conducted experiments in which we injected MI mice with siSTAT1-Cy5@FA-PNBs, OA-siSTAT1-Cy5@FA-PNBs, or siNC-Cy5@FA-PNBs as a control. After US-induced cavitation, we prepared single-cell suspensions from the anterior wall of the LV. Using flow cytometry, we sorted Cy5-positive and Cy5-negative cells from each group and then conducted qPCR to assess STAT1 expression. The results revealed a significant reduction in STAT1 expression in Cy5-positive cells from the siSTAT1-Cy5@FA-PNBs and OA-siSTAT1-Cy5@FA-PNBs groups compared with the siNC-Cy5@FA-PNBs control group. In contrast, STAT1 expression in Cy5-negative cells did not significantly differ across the groups (fig. S6, D and E). The results of confocal microscopy also revealed that in the OA-si@FA-PNBs group of mice, the number of Cx3cr1-positive cells increased, whereas the number of CD86-positive cells decreased in the border zone (Fig. 7H). However, there were no significant differences between the groups in the infarct zone and remote zone (fig. S6F). In addition, the proportion of cells double positive for Cx3cr1 and Ki67 was greater in the OA-si@FA-PNBs group than in the NC@FA-PNBs group (Fig. 7, I and J). However, there were no significant differences between the groups in the infarct zone and remote zone (fig. S6G). Compared with that in the NC@FA-PNBs, the number of cells that was positive for both CSF1R and Ki67 is also significantly greater in the MI border region in the OA-si@FA-PNBs group (Fig. 7, K and L).ELISA confirmed a significant decrease in IL-1β and TNF-α secretion (fig. S6, H and I) and an increase in IL-4 secretion in the OA-si@FA-PNBs group, along with a decrease in the secretion of TGF-β1, a key fibrosis-inducing factor (fig. S6, J and K). The proportion of cells that were positive for both CSF1R and Ki67 was also increased (fig. S6, L and M).

### OA-si@FA-PNBs improved cardiac function and decreased fibrosis in vivo

**Fig. 8.**
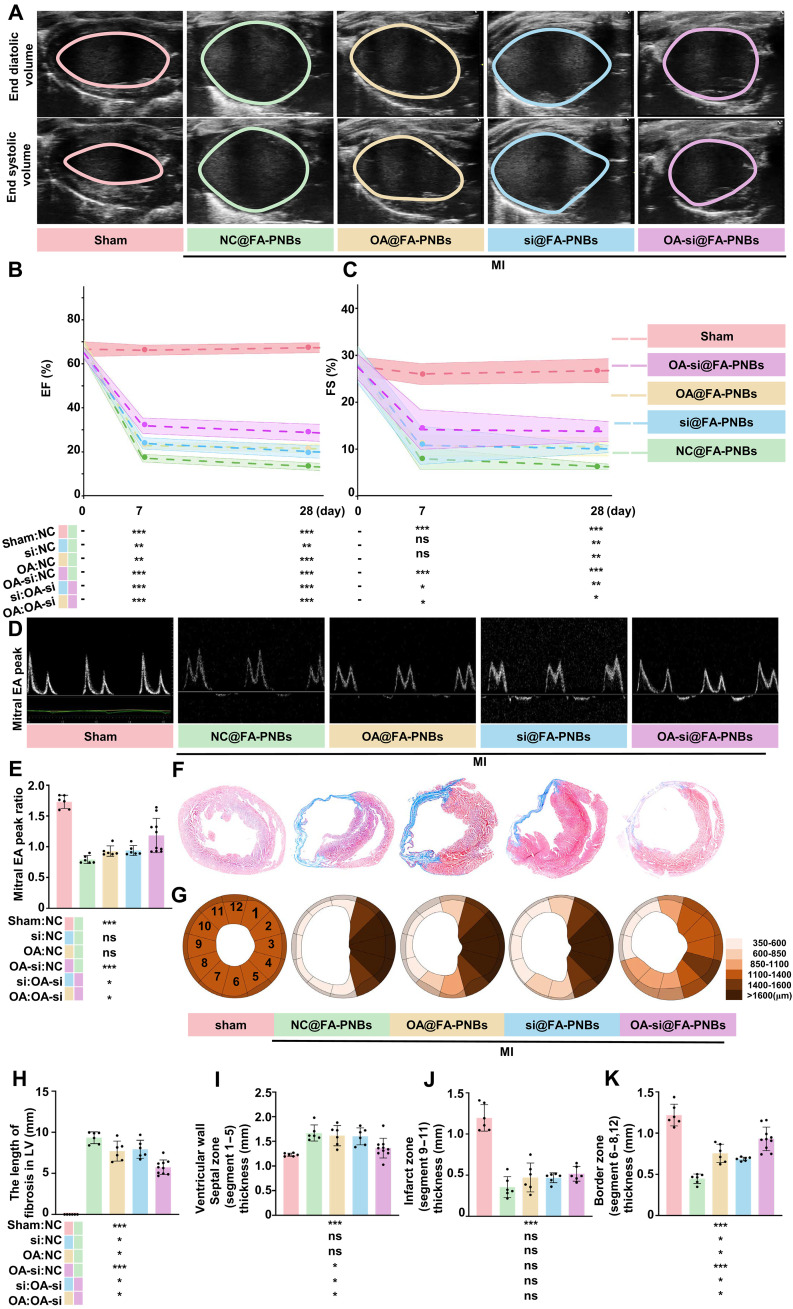
OA-si@FA-PNBs preserve cardiac function in MI model mice. (**A**) Echocardiography of the LV long-axis end diastolic/systolic volume in the treatment and sham groups 28 days post-MI. (**B** and **C**) Assessment of the LV long-axis EF and FS before MI in the treatment and MI-only groups at 7 and 28 days post-MI (sham group, *n* = 6; NC@FA-PNBs group, *n* = 6; OA@FA-PNBs group, *n* = 6; siSTAT1@FA-PNBs group, *n* = 6; OA-si@FA-PNBs group, *n* = 10). (**D**) Peak of the mitral early (E)/late (A) ventricular filling velocity in the treatment and MI-only groups 28 days post-MI. (**E**) Quantitative analysis of the mitral E/A peak ratio (sham group, *n* = 6; NC@FA-PNBs group, *n* = 6; OA@FA-PNBs groups, *n* = 6; si@FA-PNBs group, *n* = 6; OA-si@FA-PNBs group, *n* = 10). (**F**) Heart masson staining of the treatment and MI-only groups on day 28 post-MI: fibrosis area (blue) and myocytes (red). (**G**) Quantitative analysis of the LV wall thickness in 12 segments individually. (**H**) Quantitative analysis of infarct zone fibrosis length and (**I** to **K**) quantitative analysis of the LV wall thickness in the septal zone, infarct zone and border wall (sham group, *n* = 6; NC@FA-PNBs group, *n* = 6; OA@FA-PNBs group, *n* = 6; si@FA-PNBs group, *n* = 6; OA-si@FA-PNBs group, *n* = 10). Pink, the sham mice; green, MI model mice received NC@FA-PNBs via injection. Yellow, MI model mice that received OA@FA-PNBs. Blue, MI model mice that received si@FA-PNBs; purple, MI model mice received OA-si@FA-PNBs. The data are expressed as the means ± SDs. *P < 0.05, **P < 0.01, and ***P < 0.001.

The therapeutic efficacy of the different FA-PNBs was measured after MI model mice were treated with NC@FA-PNBs, si@FA-PNBs, OA@FA-PNBs, or OA-si@FA-PNBs every 3 days for 28 days. Echocardiographic assessments of LV long-axis functions, such as the ejection fraction (EF) and fractional shortening (FS) via end systolic or diastolic volume (Fig. 8A), were comparable among the sham control (Fig. 8, B and C, pink), NC@FA-PNBs (Fig. 8, B and C, green), OA@FA-PNBs (Fig. 8, B and C, yellow), si@FA-PNBs (Fig. 8, B and C, blue), and OA-si@FA-PNBs (Fig. 8, B and C, purple) groups before MI (day 0). The EFs were significantly greater in the OA-si@FA-PNBs group than in the NC@FA-PNBs, si@FA-PNBs, and OA@FA-PNBs groups (Fig. 8, B and C) at 7 and 28 days post-MI. The FS was significantly greater in the OA-si@FA-PNBs group than in the si@FA-PNBs, NC@FA-PNBs, and OA@FA-PNBs groups on day 28 post-MI (Fig. 8, B and C). The mitral E and A peak values observed on day 28 post-MI were significantly greater in the OA-si@FA-PNBs group (Fig. 8, D and E, purple) than in the NC@FA-PNBs (Fig. 8E, green), OA@FA-PNBs (Fig. 8E, yellow), and si@FA-PNBs (Fig. 8E, blue) groups. We investigated the morphology of heart samples harvested on day 28 post-MI via Masson staining (Fig. 8F). The overall degree of cardiac fibrosis observed is illustrated via a clock segment graph (Fig. 8G). Cardiac fibrosis was significantly lower in the OA-si@FA-PNBs group than in the NC@FA-PNBs group (Fig. 8H). In the border zone, the LV wall thickness was significantly greater in the OA-si@FA-PNBs group than in the NC@FA-PNBs, OA@FA-PNBs, and si@FA-PNBs groups (Fig. 8I). There was no significant difference in the LV wall thickness in the infarct zone among the NC@FA-PNBs, OA@FA-PNBs, si@FA-PNBs, and OA-si@FA-PNBs groups (Fig. 8J). In the remote zone, the LV wall thickness was significantly greater in the NC@FA-PNBs, OA@FA-PNBs, and si@FA-PNBs groups than in the OA-si@FA-PNBs group (Fig. 8K). In addition, to evaluate the effects of only US irradiation (US group), as well as the effects of the injection of free siSTAT1 and OA-NO_2_ (OA-si-NS group) and the use of Lipo 3000 to deliver siSTAT1 (lipo-OA-si group) on cardiac function, we performed cardiac US imaging 7 and 28 days post-MI. The results revealed that none of these three approaches significantly improved cardiac function (fig. S7, A to C). Masson staining further revealed that these methods did not significantly reduce the infarct size or increase the wall thickness at the infarct border zone (fig. S7, D to H). Conversely, in the OA-si@FA-PNBs group of mice, the EF and FS values were significantly higher than those in the OA-si-NS group, the lipo-OA-si group, and the US group. Furthermore, cardiac fibrosis was significantly decreased, and the border LV wall thickness was significantly greater in the OA-si@FA-PNBs group compared with the OA-si-NS group, the lipo-OA-si group, and the US group (fig. S7, D to H). To evaluate potential toxicity, hematoxylin and eosin (H&E) staining of the liver, spleen, lung, and kidney of MI model mice treated with different FA-PNBs was conducted (fig. S8A), and no significant morphological lesions were observed in any of the tissues, suggesting negligible side effects during in vivo treatment. In addition, kidney function markers, such as creatinine (CRE) and blood urea nitrogen (BUN), and liver function markers, such as aspartate aminotransferase (AST) and glutamic pyruvate transaminase (ALT), were tested in these groups (fig. S8, B to E). The measured values were all within the normal ranges, implying that the treatment had little effect on hepatic and kidney function. We also administered local cardiac injections of OA-si@FA-PNBs and included a group of mice receiving local cardiac injections of doxorubicin as a positive control. Two days later, single-cell suspensions were prepared, and flow cytometry was used to assess the proportion of apoptotic cells. Flow cytometry was performed via the Annexin V–propidium iodide assay to assess apoptotic cell populations. The results revealed no significant increase in the proportion of apoptotic cells in the hearts of the mice injected with OA-si@FA-PNBs compared with those injected with saline (fig. S8, F and G). For in vitro safety experiments, HL-1 cardiomyocytes were treated with either saline or OA-si@FA-PNBs, followed by flow cytometry analysis of apoptosis. These results suggest that OA-si@FA-PNBs did not increase the rate of apoptosis in HL-1 cells (fig. S8, H and I).

## DISCUSSION

This study reports a nanotherapeutic delivery system with CD86-positive macrophage-specific targeting and US-triggered release capabilities by leveraging nano-US contrast agents and exploiting the unique acoustic cavitation effect of US (FA-PNBs), which specifically delivers small-molecule compounds and nucleic acids to CD86-positive macrophage targets in the MI area and reduces systemic side effects. Upon the use of US-targeted NB destruction (UTND) technology in combination with LIFU, the PFH within FA-PNBs is released to produce a directional “explosion effect” that fully releases the small-molecule compound drug and nucleic acid. This approach reduces the degradation of drug molecules during blood transport, maintaining higher drug concentrations and activity within the target cells.

The innovation of this study is that we applied an US-responsive NB drug delivery system for the treatment of MI. Moreover, it was modified with FA to enhance the targeting ability of the nanodrug delivery system. The addition of PFH enables the drug delivery system to undergo a liquid-gas phase transition under LIFU irradiation in vitro to achieve accurate drug delivery. Compared with previous microbubble disruption techniques, NBs have smaller diameters, facilitating easier passage through the endothelial barrier into the infarct zone and enhancing drug accumulation and retention. This capability ensures that the drug cargos are better protected, reduces pretarget loss, improves targeting accuracy, increases the intracellular drug concentration at the target site, and enhances therapeutic outcomes while minimizing interference with nontarget cells. Through the use of biomimetic membrane technology, NBs were modified with FA to increase their infarct zone-targeting ability. Although FA is an excellent modifier that targets CD86-positive macrophages, and many studies have used FA modification as a way to target macrophages, few studies have used FA-modified nanomaterials to target drug delivery to macrophages in areas of MI. Our experimental results also demonstrate the accuracy of this approach. Our results demonstrate that the use of folate-modified nanomaterials to target macrophages in the MI area enables precise drug delivery. Subsequently, external US was applied to achieve targeted destruction of the NBs in the MI area, using unique sonoporation and cavitation effects to maximize the concentration of compound and siRNA drugs in the infarct zone. This drug delivery system is amenable for use in other inflammatory diseases characterized by the accumulation of CD86-positive immune cells or FR-positive cells in target tissues. Our results demonstrated the efficacy of the system, with OA-NO_2_ and siRNA targeting STAT1 (siSTAT1) synergistically modulating the immune environment within the MI area. This modulation not only promotes a reduction in CD86-positive macrophages and an increase in Cx3cr1-positive macrophages but also recruits T_regs_.

Another innovative point is that this nanomedicine delivery system involves both small-molecule compounds and nucleic acid therapeutics for focused treatment that is capable of targeting CD86-positive macrophages post-MI. This dual delivery mechanism facilitates the coregulation of macrophage phenotypes in situ, enabling in-depth exploration of drug interactions within a singular cellular environment. Compared with previous studies, the small-molecule compound OA-NO_2_ selected in this study can reduce the production of TGF-β1 and greatly reduce the risk of long-term cardiac fibrosis easily caused by Cx3cr1-positive macrophages. CD86-positive macrophages have been extensively studied and targeted because of their roles in disease progression and treatment. CD86-positive macrophages in the MI zone exacerbate cardiac tissue damage by releasing a high volume of proinflammatory cytokines, such as IL-1β and TNF-α, which compromise long-term patient outcomes (*25*). However, within the context of emerging nanotherapeutics in clinical settings for the treatment of MI, treatments targeting CD86-positive macrophages in MI are scarce.

Many studies have aimed to induce the transition from the M1 macrophage phenotype to the CD206-positive M2 phenotype. However, the CD206-positive M2 phenotype releases excessive amounts of TGF-β1, which activates myofibroblasts, leading to myocardial fibrosis (*26*). Previous cell therapy studies used Cx3cr1-positive macrophages to inhibit the inflammatory response after MI to repair ischemic heart damage (*9*), and we found that this group of cells secreted a decrease in TGF-β1, which helped improve long-term cardiac function.

In this study, we used a combination of OA-NO_2_ and siSTAT1 to treat CD86-positive macrophages in the MI area. After 4 days of treatment, we observed a decrease in the number of CD86-positive cells and an increase in the number of Cx3cr1-positive cells. On the basis of our in vitro results, we hypothesize that OA-NO_2_ and siSTAT1 together induce the conversion of CD86-positive macrophages into Cx3cr1-positive macrophages. In addition, our results revealed increased expression of CSF1R in Cx3cr1-positive cells in the treatment group, which may have led to the proliferation of Cx3cr1-positive macrophages, which may have contributed to the increased number of Cx3cr1-positive cells.

Our nanomedicine delivery platform represents a breakthrough in targeted therapeutics for MI, merging precision targeting with the simultaneous conveyance of compound- and nucleic acid–based treatments. We developed a highly efficient nanomedicine delivery system carrying both compound and nucleic acid therapeutics for focused treatment that is capable of targeting CD86-positive macrophages post-MI. Leveraging US-activated NBs encapsulating OA-NO_2_ and siSTAT1, our system shows exceptional cargo release control, ensuring enhanced targeting fidelity and therapeutic concentration at the site of action. This multimolecular delivery system facilitates the coregulation of macrophage phenotypes in situ, enabling in-depth exploration of drug interactions within a singular cellular environment.

In particular, concerted modulation by OA-NO_2_ and siSTAT1 ameliorates the post-MI immune milieu. The resulting attenuation of adverse ventricular remodeling and preservation of cardiac functionality underscore the system’s translational promise, heralding a novel approach for integrative immune environment regulation therapy post-MI with profound clinical implications. Despite the discovery of a novel FA-PNB drug delivery system and the molecular drug combination of OA-NO_2_ and siSTAT1, several limitations warrant further investigation. First, during the preparation of cardiac single-cell suspensions, some cells, particularly cardiomyocytes, may have been lost, which could have affected our results. Our single-cell sequencing results initially revealed that the primary target of FA-PNBs was CD86-positive cells, predominantly macrophages but also monocytes and dendritic cells. This observation is consistent with previous research and may be related to the nature of immune cell recognition of exogenous substances (*27*). In addition, the small-molecule compound used in this study was not selected through high-throughput screening, suggesting that there may be more effective combinations for modulating macrophage function. This presents a potential direction for future research. Moreover, while our study revealed an increase in the number of Cx3cr1-positive macrophages in the MI area, we lack definitive evidence on whether these cells result from the transformation of other cell types, self-proliferation, or migration from other organs. Future studies using lineage tracing methods could provide deeper insights. Last, to further validate the role of Cx3cr1-positive macrophages in regulating the post-MI immune environment, future research should use Cx3cr1 conditional knockout transgenic mice. In conclusion, FA-PNBs not only represent a highly promising drug delivery approach for modulating the immune microenvironment in MI but also provide a more precise tool for studying the interactions of two molecules within the same cell population.

## MATERIALS AND METHODS

### Experimental design

This study was performed to evaluate the efficacy and targeting of a nanotherapeutic delivery system with CD86-positive macrophage-specific targeting and US-triggered release capabilities. This objective was addressed by (i) developing a FA-modified US-responsive gene/drug delivery system based on the self-assembly of 1,2-dioleate-3-trimethylammonium propane (DOTAP), 1,2-dibutyryl-sn-glucose-3-phosphateethanolamine-N-[folate (polyethylene glycol–2000] (DSPE-PEG2000-FA), cholesterol, and PFH, namely, FA-PNBs; (ii) studying the transfection efficiency and targeting of FA-PNBs to CD86-positive macrophages in the heart infarct zone; (iii) exploring the synergistic regulation of the repolarization of CD86-positive macrophages into small molecule compounds (OA-NO_2_) and nucleic acid drugs (siSTAT1) carried by this delivery system; and (iv) determining that this system modulates the immune microenvironment and improves cardiac function and decreases fibrosis in MI model mice, (v) indicating that there are no signs of toxicity upon MI treatment via tail vein injection of OA-si@FA-PNBs. The researchers used a blinded method during the experiment.

### Materials

1,2-Distearoyl-*sn*-glycerol-3-phosphateethanolamine-*N*-[methoxy (polyethylene glycol)–2000] (DSPE-PEG2000), DSPE-PEG2000-FA, cholesterol, and DOTAP were purchased from Xi’an Ruixi Biotechnology (Xi’an, China). PFH was purchased from C. Zihai (Chongqing, China). OA-NO_2_ was purchased from Cayman (Wuhan, China). Fetal bovine serum (FBS), cell culture medium (DMEM), and Opti-MEM were obtained from Gibco BRL (Grand Island, USA). Agarose, CCK-8, Hoechst, trypsin, and serum-free cell freezing media were obtained from Biosharp (Anhui, China). LPS was purchased from Sigma-Aldrich (USA). TRIzol and SYBR Green PCR Master Mix were purchased from Tiangen (Beijing, China). The double-stranded siRNAs targeting STAT1 (siSTAT1) and Cy5-siSTAT1 used to knock down STAT1 gene expression in the study were obtained from GenePharma (Shanghai, China). The sequences of the siRNAs are shown in table S1. The primers used were obtained from RuiBiotech (Beijing, China), and their specific sequences can be found in table S2.

### Cell culture

RAW264.7 macrophages and immortalized mouse embryonic fibroblasts (NIH3T3 cells) were procured from the central laboratory of Peking University Third Hospital. Mouse cardiomyocytes (HL-1 cells) were obtained from FuHeng Biology (Shanghai, China). RAW264.7 and NIH3T3 cells were cultured in Dulbecco’s modified Eagle’s medium (DMEM) supplemented with 10% FBS and maintained at 37°C in a 5% CO_2_ atmosphere. HL-1 cells were cultivated in modified Eagle’s medium (MEM) supplemented with 10% FBS under identical conditions. To induce CD86-positive macrophage polarization, RAW264.7 cells in DMEM containing 10% FBS were treated with LPSs (1 μg/ml; Sigma-Aldrich) for 24 hours.

### Liposome preparation

A FA-modified US-responsive gene/drug delivery system, termed the FA-PNBs, was formulated using DOTAP, DSPE-PEG2000-FA, cholesterol, and PFH. FA-PNBs were synthesized via a standard thin-film hydration method. DOTAP, cholesterol, and DSPE-PEG2000-FA were dissolved in trichloromethane in a round-bottom flask at a molar ratio of 3:1:1. OA-NO_2_ nanoparticles were dissolved in trichloromethane and then added to the lipid mixture at 25% of the lipid weight. The mixture was evaporated via a rotary evaporator at 100 mbar and 120 rpm for 15 min at 55°C. Subsequent evaporation at 0 mbar for an additional hour completed solvent removal and resulted in the formation of a thin lipid film. This film was hydrated with 4 ml of PBS (0.01 M, pH 7.4) and sonicated at 60°C for an hour. Following the addition of 200 μl of PFH, the suspension was emulsified via a sonicator at 125 W in an ice-water bath, with sonication intervals of 5 s and rest periods of 5 s for 8 min in total. The nanoemulsion was then centrifuged at 5000*g* for 5 min at 4°C, and the supernatant was discarded. This process was repeated three times to eliminate free lipids and excess reactants. Vesicles were collected via mini extrusion through a 100-nm membrane and further purified via dialysis. The resulting vesicles were stored at 4°C for future use. Positively charged FA-PNBs were mixed with siSTAT1 solution in enzyme-free water and incubated at room temperature for 10 min to bind siSTAT1 to FA-PNBs to generate si@FA-PNBs. FA-PNBs loaded with OA-NO_2_ are denoted as OA@FA-PNBs; those conjugated with siSTAT1 are denoted as si@FA-PNBs. OA-si@FA-PNBs indicate FA-PNBs simultaneously carrying OA-NO_2_ and siSTAT1. The NC@FA-PNBs served as empty nanocarriers for the control group.

### Characterization of FA-PNBs

Dynamic light scattering (Nano-ZS, Malvern) was used to assess the particle size and surface potential of the FA-PNBs, including the NC@FA-PNBs, si@FA-PNBs, OA@FA-PNBs, and OA-si@FA-PNBs. The stability of the OA-si@FA-PNB nanocarriers was monitored through hydromechanical particle size measurements on days 1, 3, 5, 7, and 10. For structural and morphological examination, droplets of diluted nanoparticle solution were placed on copper mesh and silicon wafers and then dried at room temperature for TEM analysis. The encapsulation efficiency and loading capacity of OA-NO_2_ were calculated via the following formulas: encapsulation efficiency (%) = (mass of OA-NO_2_/total OA-NO_2_) × 100; loading capacity (%) = (mass of OA-NO_2_/total liposomes) × 100.

### Gel retardation assay

To assess the ability of FA-PNBs to carry siSTAT1, agarose gel electrophoresis was performed. siSTAT1 was incubated with FA-PNBs at various mass ratios (1:0 to 1:30) for 10 min at room temperature. After incubation, 6× loading buffer was added, and the samples were subjected to electrophoresis on a 3% agarose gel at 100 V for 30 min. Pretreatment of all the instruments with enzyme-free water ensured contamination-free conditions. After electrophoresis, the gel was imaged to observe the band shifts of siSTAT1.

### Cytotoxicity assay

RAW264.7 macrophages and HL-1 cells were seeded onto 96-well plates at a density of 1 × 10^5^ cells/cm^2^ for 24 hours. The cells were then treated with PBS, NC@FA-PNBs, OA@FA-PNBs, si@FA-PNBs, or OA-si@FA-PNBs at concentrations ranging from 0 to 400 μg/ml for 24 hours. After incubation, the cells were washed with PBS and treated with CCK-8 reagent for 4 hours, and the absorbance was measured at 450 nm.

### US imaging in vitro

The phase transition behavior of FA-PNBs and the role of PFH were examined under LIFU (1.0 MHz, focal length of 1.5 cm, duty cycle of 50%, pulse-wave mode, Chongqing Medical University, China) at various acoustic intensities (1 to 4 W/cm^2^) and a frequency of 1.0 MHz. The duration of US irradiation varied from 1 to 4 min, and optical microscopy was used to observe changes. A high-frequency linear array probe and US system (Esaote MyLab90, Florence, Italy) was used, and images were captured in B-mode and contrast-enhanced US modes, with preirradiation images serving as controls. US imaging software (FDY-II, Institute of Ultrasound Imaging, Chongqing, China) was used for image analysis.

### Measurement of FRβ expression and cell binding assays

Cardiomyocytes (HL-1 cells), fibroblasts (NIH3T3 cells), CD86(−) macrophages (RAW264.7 cells), and CD86(+) macrophages were seeded onto 6-well plates at a density of 1 × 10^5^/cm^2^ and cultured in FA-free DMEM. After the cells had attached to the plate, they were collected and resuspended in 500 μl of PBS. For analysis of cells collected from in vitro experiments, the cells were incubated for 10 min in TruStain FcX (1:200; BioLegend, catalog no. 101320) to block Fc receptors, incubated with phycoerythrin (PE)–conjugated anti-mouse FRβ (for detailed information on the antibodies, see table S3) for 15 min at 4°C and then washed twice. The fluorescence intensity was analyzed via flow cytometry (CytoFLEX, Beckman Coulter, USA), with 10,000 events collected.

### Evaluation of transfection efficiency

#### Flow cytometry

RAW264.7 cells were seeded onto six-well plates at a density of 1 × 10^5^ cells/cm^2^ and activated with LPS (1 μg/ml) for 24 hours. siSTAT1 was labeled with Cy5. Then, PBS, Lipo3000-Cy5-siSTAT1, Cy5-si@PNBs, or Cy5-si@FA-PNBs were added to the cell culture medium. In addition, a group of cells received Cy5-si@FA-PNBs and LIFU at a power density of 3 W/cm^2^ for 3 min. After 6 hours, the cells were collected. The fluorescence intensity was analyzed via flow cytometry (CytoFLEX, Beckman Coulter, USA), with 10,000 events collected.

#### Immunofluorescence assay

RAW264.7 cells were seeded onto six-well plates at a density of 1 × 10^5^ cells/cm^2^ and activated with LPS (1 μg/ml) for 24 hours. siSTAT1 was labeled with Cy5. Then, PBS, Lipo3000-Cy5-siSTAT1 (lipo), Cy5-si@PNBs (PNBs), and Cy5-si@FA-PNBs (FA-PNBs) were added to the cell culture medium. In addition, a group of cells was treated with Cy5-si@FA-PNBs and LIFU at a power density of 3 W/cm^2^ for 3 min (FA-PNBs + US). After 3 days, the medium was removed, the cells were washed twice with PBS, Hoechst dye was added, and the cells were incubated at room temperature for 15 min. Last, the plates were sealed and observed via confocal laser scanning microscopy.

### Evaluation of the ability of nanomaterials to modulate the macrophage phenotype

#### Flow cytometry

This study used CD86 as a specific marker for CD86-positive macrophages and CX3C chemokine receptor 1 (Cx3cr1) for reparative Cx3cr1-positive macrophages. Following the induction of CD86-positive macrophages, the cells were treated with NC@FA-PNBs, OA@FA-PNBs, si@FA-PNBs, or OA-si@FA-PNBs, and all the groups received US stimulation. After 3 days, the cells were collected and resuspended in PBS for flow cytometry analysis with anti-F4/80, anti-CD11b, anti-CD86, and anti-Cx3cr1 antibodies (for detailed information on the antibodies, see table S3), and the data were analyzed via Kaluza software (Beckman Coulter).

#### Immunofluorescence assay

After inducing macrophages to become CD86-positive macrophages, the cells were incubated with NC@FA-PNBs, OA@FA-PNBs, si@FA-PNBs, or OA-si@FA-PNBs. Three days later, the supernatant was removed, and the cells were fixed with 4% paraformaldehyde for 30 min. Then, the cells were incubated with primary antibodies overnight at 4°C and then incubated with the respective fluorescently labeled secondary antibodies for 1 hour. The nuclei were stained with 4′,6-diamidino-2-phenylindole (DAPI), and the cells were mounted onto slides. The samples were observed with a fluorescence microscope. The primary antibodies used were anti-CD86 and anti-CD11b (for detailed information on the antibodies, see table S3). The secondary antibodies used were Alexa Fluor 488–conjugated goat anti-rat immunoglobulin G (IgG) (1:1000; Thermo Fisher Scientific, A48262) and Alexa Fluor 555–conjugated goat anti-rabbit IgG (1:1000; Thermo Fisher Scientific, A32732).

#### Enzyme-linked immunosorbent assay

The concentrations of mouse IL-1β (Elabscience, #E-EL-M0037), TNF-α (Elabscience, #E-EL-M3063), IL-4 (Elabscience, #E-EL-M0043), and TGF-β1 (Elabscience, #E-UNEL-M0099) in the cell culture supernatants were determined via ELISA, as per the manufacturer’s guidelines.

### Transcriptomic analysis

RAW264.7 cells were seeded onto six-well plates at a density of 1 × 10^5^ cells/cm^2^ and activated with LPS (1 μg/ml) for 24 hours. Then, NC@FA-PNBs, OA@FA-PNBs, si@FA-PNBs, or OA-si@FA-PNBs (200 μg/ml) were added. After 72 hours, the cells were harvested. Total RNA was isolated from tissues via a TRIzol kit (Invitrogen), and RNA integrity was assessed via an Agilent 2200 TapeStation (Agilent Technologies, USA). The libraries that passed the quality test were sequenced via the Illumina platform (sequencing strategy PE150) to measure mRNA expression. The DESeq2 package was used for differential gene expression analysis. Differentially expressed genes (DEGs) were identified via the following criteria: |log_2_fold change| ≥ 1.5 and *q* (*P* after correction) < 0.05. GO and KEGG analyses were used to analyze the enriched DEGs.

### Quantification of inflammation-related gene expression via qRT-PCR

For qRT-PCR quantification, RAW264.7 cells were seeded onto six-well plates at a density of 1 × 10^5^ cells/cm^2^ and activated with LPS (1 μg/ml) for 24 hours. Then, NC@FA-PNBs, OA@FA-PNBs, si@FA-PNBs, or OA-si@FA-PNBs (200 μg/ml) were added. After 72 hours, the cells were harvested, and total RNA was extracted via TRIzol reagent (Tiangen, Beijing, China). cDNA was generated via reverse transcription with a High-Capacity cDNA Reverse Transcription Kit (Yeasen, Shanghai, China) according to the manufacturer’s instructions. Gene expression was assessed via SYBR Green qRT-PCR (Yeasen, Shanghai, China), and target gene expression was compared between samples via normalization to β-actin expression and application of the 2^−∆∆*C*t^ formula. The primer sequences are listed in the table S2.

### Acute MI mouse model

All experimental procedures and protocols were approved by the Peking University Third Hospital Committee on Ethics for the Care and Use of Laboratory Animals (no. A2023109). Institute of Cancer Research (ICR) mice were purchased from the Department of Laboratory Animal Science of Peking University Health Science Center (Beijing, China) and maintained on standard chow and water.

Adult Institute of Cancer Research (ICR) mice (male, 14 to 16 weeks) were used for in vivo experiments. The mice were housed in groups at 22° to 24°C and a relative humidity of 30 to 70% with free access to food and water and maintained under a 12-hour light/12-hour dark cycle. A model of MI was induced by permanent ligation of the left coronary artery, as previously described by our group (*28*).

### In vivo biodistribution of the nanoparticles

The histological distribution of Cy5-labeled Cy5-siSTAT1@FA-PNBs or Cy5-siSTAT1@PNBs was monitored 3 days after MI via an IVIS Lumina XRMS Series III imaging system (Caliper Life Sciences). All the mice were fed an alfalfa-free diet to reduce background fluorescence. Cy5-si@FA-PNBs or Cy5-si@PNBs were intravenously injected into MI mice. At 1, 2, 4, 8, 16, 24, and 48 hours after injection, the fluorescence biodistribution of Cy5 in the heart was analyzed. Then, the treated mice were anesthetized with 2.5% isoflurane, and the main organs (heart, liver, spleen, lung, and kidney) were collected and subjected to ex vivo imaging of Cy5 fluorescence.

### Macrophage phenotype transition study in the MI area

We initiated the corresponding intervention treatment 3 days after establishing the MI mouse model and administered treatments every 3 days. On the 7th day post-MI, samples were collected for experiments, including immunofluorescence and flow cytometry.

#### Flow cytometry

The portion below the ligation line was cut and made into a single-cell suspension following the manufacturer’s guidelines (Miltenyi Biotec, # 130-110-204). The single-cell suspension was filtered through a 70-μm filter, and agents were added to lyse the red blood cells and remove tissue debris. A single-cell suspension obtained after centrifugation was used to analyze macrophages via flow cytometry. To analyze the frequency of T_regs_ (CD4^+^Foxp3^+^), single-cell suspensions were stained with anti-CD4 and anti-Foxp3 antibodies according to the manufacturer’s protocols. We also analyzed Cx3cr1-positive macrophages (F4/80, CD11b, CD206, and Cx3cr1) and CD86-positive macrophages (F4/80, CD11b, and CD86) in heart tissue cell suspensions stained with anti-F4/80-PE, anti-CD11b, anti-86, and anti-Cx3cr1 antibodies (for detailed information on the antibodies, see table S3) via flow cytometric analysis.

#### Immunofluorescence assay

After fixation in paraformaldehyde overnight at 4°C, the myocardial tissue was cut into serial frozen coronal slices with a thickness of 10 μm. First, the frozen sections were rinsed with PBS, the cell membranes were disrupted with 0.3% Triton X-100, and then the sections were blocked with serum. Second, all slices were incubated overnight with primary antibody in a 4°C refrigerator. Primary antibodies against CD86, Cx3cr1, Ki67, and CSF1R were used (for detailed information on the antibodies, see table S3). Then, the slices were incubated with the secondary antibodies Alexa Fluor 488–conjugated goat anti-rat IgG (1:1000; Thermo Fisher Scientific, A48262) and Alexa Fluor 555-conjugated goat anti-rabbit IgG (1:1000, Thermo Fisher Scientific, A32732) for 1 hour. Last, the slices were counterstained with DAPI, and images were acquired with a fluorescence microscope (Olympus, Tokyo, Japan). Image-Pro Plus 6.0 (Media Cybernetics, San Diego, CA, USA) was used for the subsequent analyses.

### Evaluation of cardiac function and fibrosis

#### Masson’s staining

The hearts of the mice with MI were fixed overnight in 4% paraformaldehyde, frozen sections (10 μm) were cut, routine Masson staining was performed according to the Masson kit procedure, and the sections were photographed under an optical microscope (Model IX71 Olympus, Tokyo, Japan). The MI zone fibrosis length (blue area) and LV wall thickness were measured via NPD View version 2.6.8 (Hamamatsu, Japan). The LV sections were divided into 12 areas per section, with areas 1 to 5 corresponding to the septal wall and areas 6 to 12 to the lateral wall. Segments 9 to 11 were labeled the MI zone, and segments 6 to 8 and 12 were labeled the border zone.

#### Echocardiography

The mice underwent cardiac US tests before modeling, 7 days after modeling, and 28 days after modeling to assess heart function. After anesthesia via 2.5% isoflurane inhalation and depilation of the thoracic area, we performed parasternal B-ultrasonography via echocardiography and a high-resolution imaging system with a 40-MHz high-frequency probe (Vevo 3100 Imaging System, FUJIFILM VisualSonics Inc., USA). Each test lasted 5 to 10 cycles. The main measures used were the LVEF, FS, and mitral valve E/A peak, which were measured by double-blind operators via Vevo LAB analysis software (FUJIFILM VisualSonics, Inc.).

### Systemic toxicity evaluation

The toxicity of the nanomaterials to the liver, spleen, lung, and kidney was evaluated via H&E staining. The livers, spleens, lungs, and kidneys of the mice were fixed in 4% paraformaldehyde overnight, and paraffin sections (10 μm) were prepared. The slides were examined via H&E staining according to the manufacturer’s instructions (Beyotime Institute of Biotechnology, C0105S). The slices were observed under an optical microscope (Model IX71 Olympus, Tokyo, Japan). Serum levels ALT, AST, CRE, and BUN were measured via a HITACHI 7100 automatic biochemical analyzer.

### Single-cell RNA-seq

We induced two MI mice and administered OA-si-Cy5@FA-PNBs via tail vein injection 3 days after MI. At 7 days post-MI, we harvested myocardial tissue (mainly from the LV anterior wall) to prepare a single-cell suspension. We then sorted Cy5-positive and Cy5-negative cells via flow cytometry. The Cy5-positive cells from both groups of mice were pooled together, as were the Cy5-negative cells. Ultimately, we obtained two tubes of cells, one containing Cy5-positive cells and the other containing Cy5-negative cells.

Single-cell suspensions were prepared and assessed for quality control. Only samples with a cell viability greater than 85% and a concentration between 700 and 1200 cells/μl were used. The prepared single-cell suspension was mixed with gel beads and droplets and introduced into the different channels of a Chromium Chip G. A microfluidic device facilitated the encapsulation of individual cells within gel bead–encapsulated microdroplets (GEMs). In GEMs, the dissolution of gel beads releases barcoded primer sequences, whereas cellular lysis releases mRNA. Reverse transcription was conducted to convert mRNA into cDNA, incorporating 10x barcodes and unique molecular identifiers (UMIs). Following GEM rupture, single-stranded cDNA was purified via magnetic beads and subjected to PCR amplification to produce double-stranded cDNA. The cDNA was fragmented enzymatically into 200–to 300–base pair pieces, followed by end repair, A-tailing, and adapter ligation with Read2 sequencing primers. This process resulted in the construction of a 10X 3′ transcriptome library containing P5 and P7 adapters and dual-end indices. The quality-checked libraries were sequenced via an Illumina platform, with sequencing conducted by Annoroad.

FASTQ files of 2 samples (Cy5-positive cells and Cy5-negative cells) were processed via the Cell Ranger (v. 7.1.0) counting pipeline coupled with the mouse reference version Mus_musculus.GRC m38 to generate feature-barcode matrices. First, we filtered cells that were predicted as double cells via the use of a scrublet and DoubletFinder software. Next, the Seurat object was generated via the Seurat package with R software with the following criteria: (i) min. cells = 3, (ii) 200 < nFeature_RNA < 10,000, and (iii) percent.mt < 0.2. In other words, genes expressed in at least two cells and cells with gene numbers ranging from 200 to 10,000 were retained for further analysis. Low-quality cells were also filtered if ≥20% UMIs were derived from the mitochondrial genome. Double-cell suspensions were filtered.

After filtering, 24,939 cells (Cy5-positive samples: 12,950; Cy5-negative samples: 11,989) were retained for downstream analysis, with a median UMI of 4720 and a gene number of 1926 in Cy5-positive samples and a median UMI of 4118 and a gene number of 1979 in Cy5-negative samples. All samples were further integrated following “tips for integrating large datasets” pipeline of Seurat or using the “RunFastMNN” function in the Seurat-Wrappers package. The raw counts were normalized using 12,000 as the scale factor per cell and log (count+1) transformed. The top 2000 highly variable genes were identified for integration. Data scaling and principal components analysis were performed with default settings. Then, the significant principal components (PCs) were selected on the basis of the elbow of the standard deviations of the PCs and used for neighbor detection, followed by Louvain clustering and uniform manifold approximation and projection (UMAP). The same analysis pipeline was used for subcluster detection.

### Statistical analysis

GraphPad Prism 8.0 (GraphPad Software, La Jolla, USA) was used for statistical analysis. The data are shown as the means ± SDs. The normal distribution was detected via the Shapiro-Wilk test. For normally distributed data, ordinary one-way analysis of variance (ANOVA) with Sidak’s post hoc test was performed. When the data were not normally distributed, Brown-Forsythe and Welch-ANOVA followed by an unpaired *t* test with Welch’s correction were performed. All the results of the in vitro experiments were analyzed via Brown-Forsythe and Welch ANOVA followed by an unpaired *t* test with Welch’s correction. Differences were considered statistically significant at *P* < 0.05; *P < 0.05, **P < 0.01, and ***P < 0.001, no significance (ns).
