# Supplementary material for: Treating myocardial infarction via a nano-ultrasonic contrast agent-mediated high-efficiency drug delivery system targeting macrophages
Source: Sci Adv. 2025 Jan 3;11(1):eadp7126. doi: 10.1126/sciadv.adp7126 (PMC11698097; doi:10.1126/sciadv.adp7126)
Supplement: Supplementary file 1 — Figs. S1 to S8 Tables S1 to S3 [file sciadv.adp7126_sm.pdf]

Science Advances
AAAS

Supplementary Materials for

Treating myocardial infarction via a nano-ultrasonic contrast agent-mediated high-efficiency drug delivery system targeting macrophages

Zhen Ma *et al.*

Corresponding author: Rui Guo, rui_guo@pku.edu.cn; Yingying Zhang, zhangyy@xzhmu.edu.cn; Yunpeng Ling, micsling@pku.edu.cn

Sci. Adv. **11**, eadp7126 (2025)
DOI: 10.1126/sciadv.adp7126

This PDF file includes:

Figs. S1 to S8
Tables S1 to S3

Supplementary Figures

Fig. S1

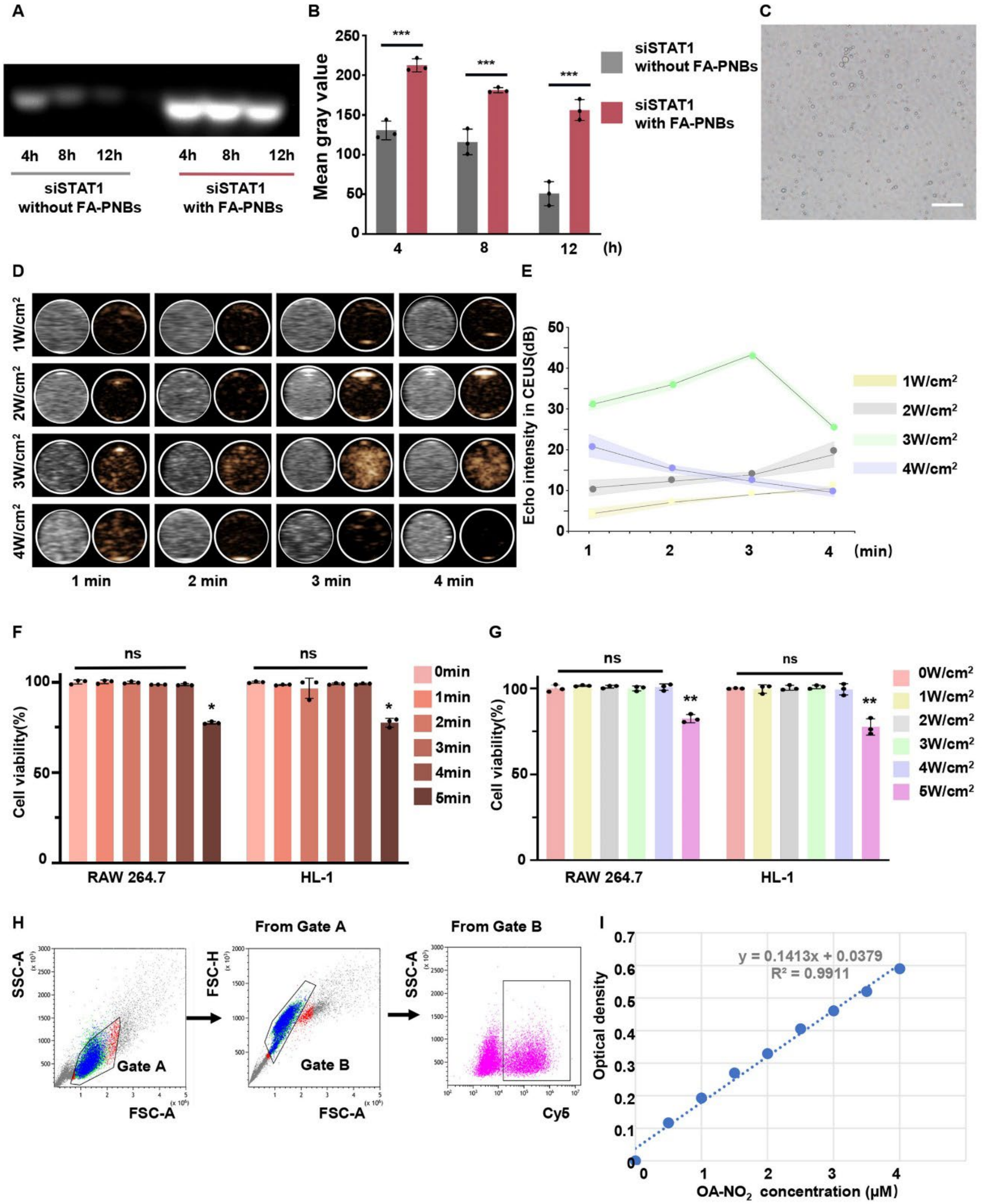

Fig. S1. (A) Agarose gel electrophoresis analysis of the stability of FA-PNBs loaded with siRNA in serum. (B) Quantitative analysis of the average fluorescence intensity of the agarose gel bands. (C) Phase change microscopy image of FA-PNBs (scale bar = 20 μm). (D) Ultrasound imaging (left: B-Mode, right: CEUS) of FA-PNBs showing time and intensity-dependent

acoustic droplet vaporization (ADV). (E) Quantitative analysis of the echo intensity of FA-PNBs after LIFU irradiation at different intensities and times in B-mode and CEUS-mode. (F) and (G) Effects of different ultrasound parameters on the viability of HL-1 cells and RAW264.7 cells. *Compared with control group. (H) Representative gating strategy for flow cytometry analysis of Cy5-tagged siSTAT1. (I) The OA-NO_2 standard curves as determined by atomic absorption spectroscopy. OA: nitro-oleic acid. The data are expressed as means ± SD, n = 3 per group. *P<0.05, **P<0.01, ***P<0.001, ns: no significance.

Fig. S2

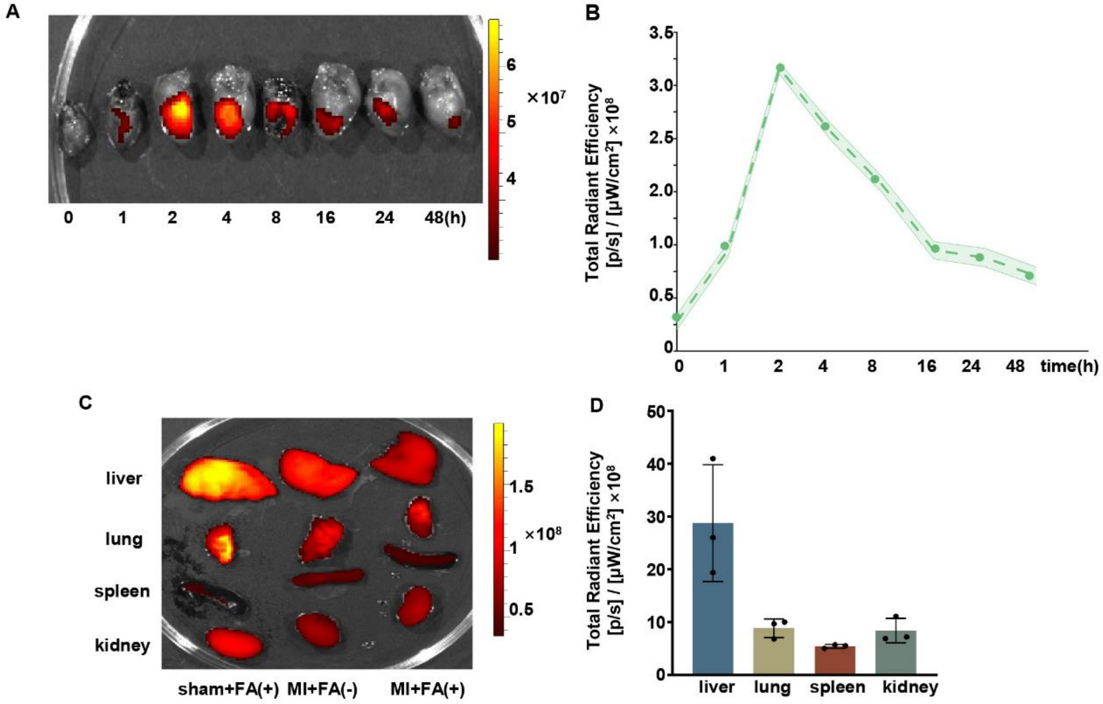

Fig. S2. (A) Ex vivo optical imaging and (B) quantitative fluorescence intensity of siSTAT1-Cy5 accumulation in the heart region of MI mice at different times post-tail vein injection, n=3 per group. (C) Ex vivo optical imaging and (D) quantitative fluorescence intensity of major organs (liver, spleen, lung, and kidney) examined 2 hours post-injection, n=3 per group.

Fig. S3

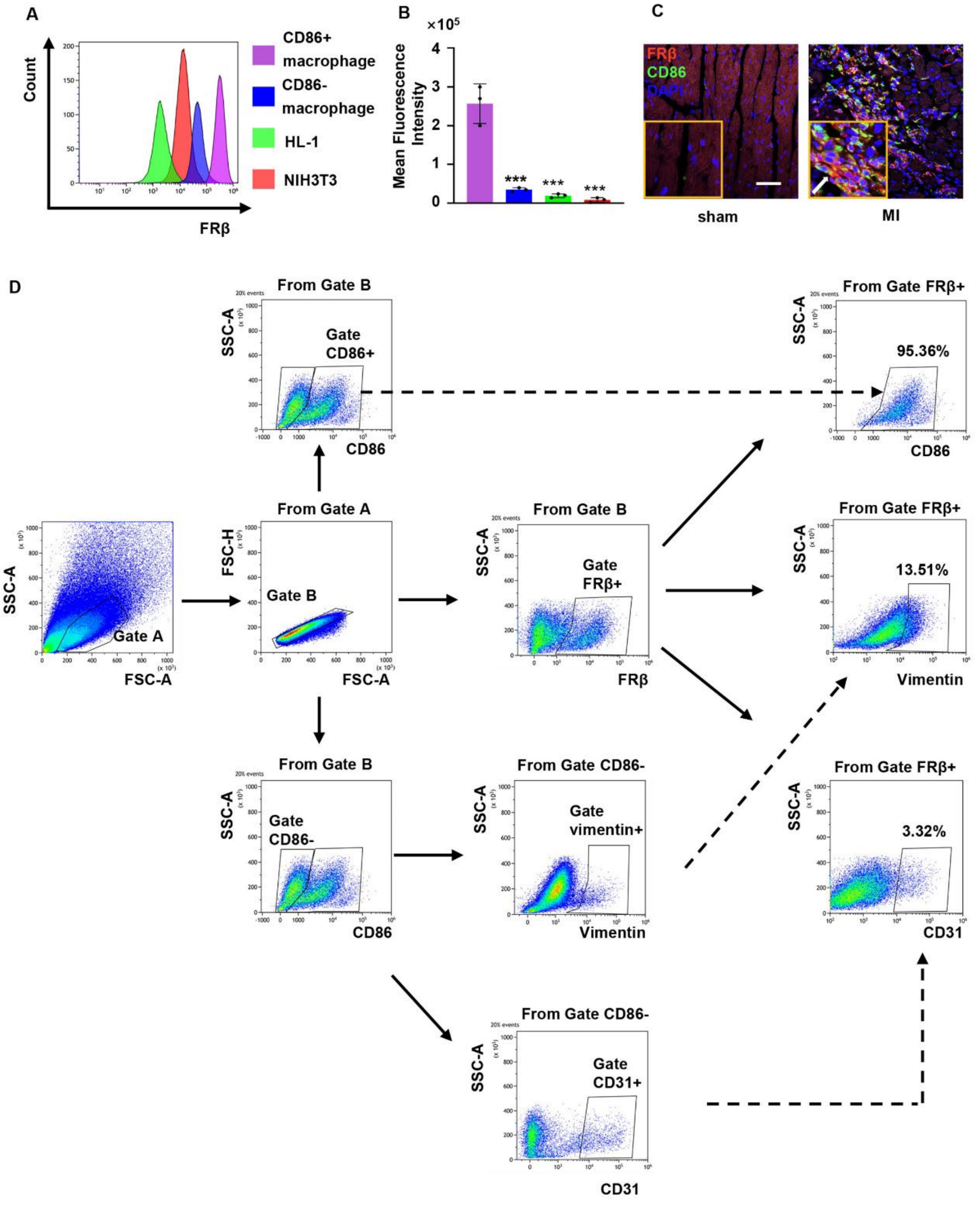
A
Count
FRβ
CD86+ macrophage
CD86- macrophage
HL-1
NIH3T3
B
×10^5
Mean Fluorescence Intensity
***
C
FRβ
CD86
DAPI
sham
MI
D
From Gate B
Gate CD86+
From Gate FRβ+
95.36%
From Gate A
Gate A
Gate B
Gate FRβ+
13.51%
Gate CD86-
From Gate CD86-
Gate vimentin+
3.32%
Gate CD31+
SSC-A
FSC-A
FSC-H
CD86
FRβ
Vimentin
CD31

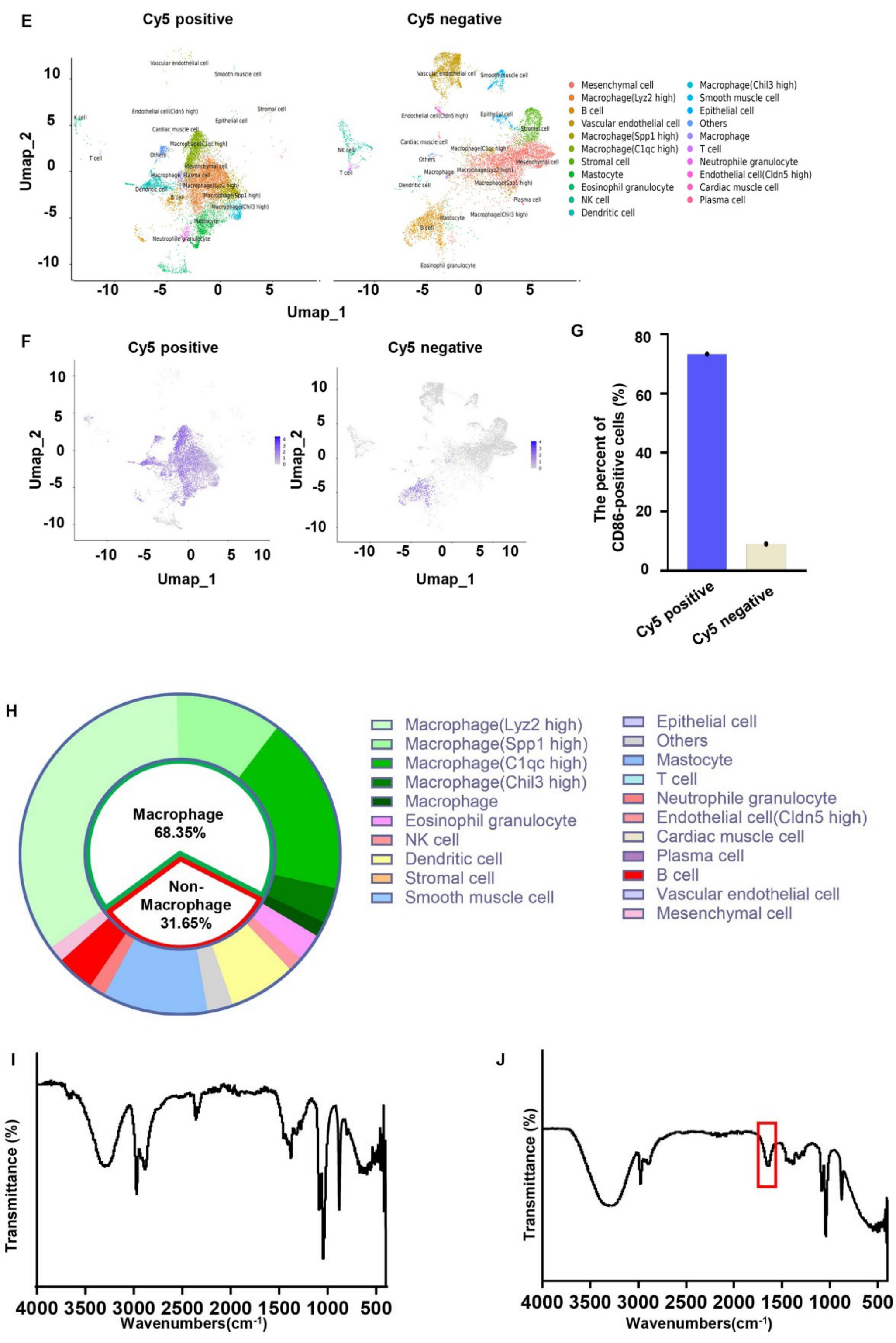
E
Cy5 positive
Cy5 negative
Umap_2
Umap_1
Mesenchymal cell
Macrophage(Lyz2 high)
B cell
Vascular endothelial cell
Macrophage(Spp1 high)
Macrophage(C1qc high)
Stromal cell
Mastocyte
Eosinophil granulocyte
NK cell
Dendritic cell
Macrophage(Chil3 high)
Smooth muscle cell
Epithelial cell
Others
Macrophage
T cell
Neutrophile granulocyte
Endothelial cell(Cldn5 high)
Cardiac muscle cell
Plasma cell
F
Cy5 positive
Cy5 negative
Umap_2
Umap_1
G
The percent of CD86-positive cells (%)
Cy5 positive
Cy5 negative
H
Macrophage 68.35%
Non-Macrophage 31.65%
Macrophage(Lyz2 high)
Macrophage(Spp1 high)
Macrophage(C1qc high)
Macrophage(Chil3 high)
Macrophage
Eosinophil granulocyte
NK cell
Dendritic cell
Stromal cell
Smooth muscle cell
Epithelial cell
Others
Mastocyte
T cell
Neutrophile granulocyte
Endothelial cell(Cldn5 high)
Cardiac muscle cell
Plasma cell
B cell
Vascular endothelial cell
Mesenchymal cell
I
Transmittance (%)
Wavenumbers(cm-1)
J
Transmittance (%)
Wavenumbers(cm-1)

K

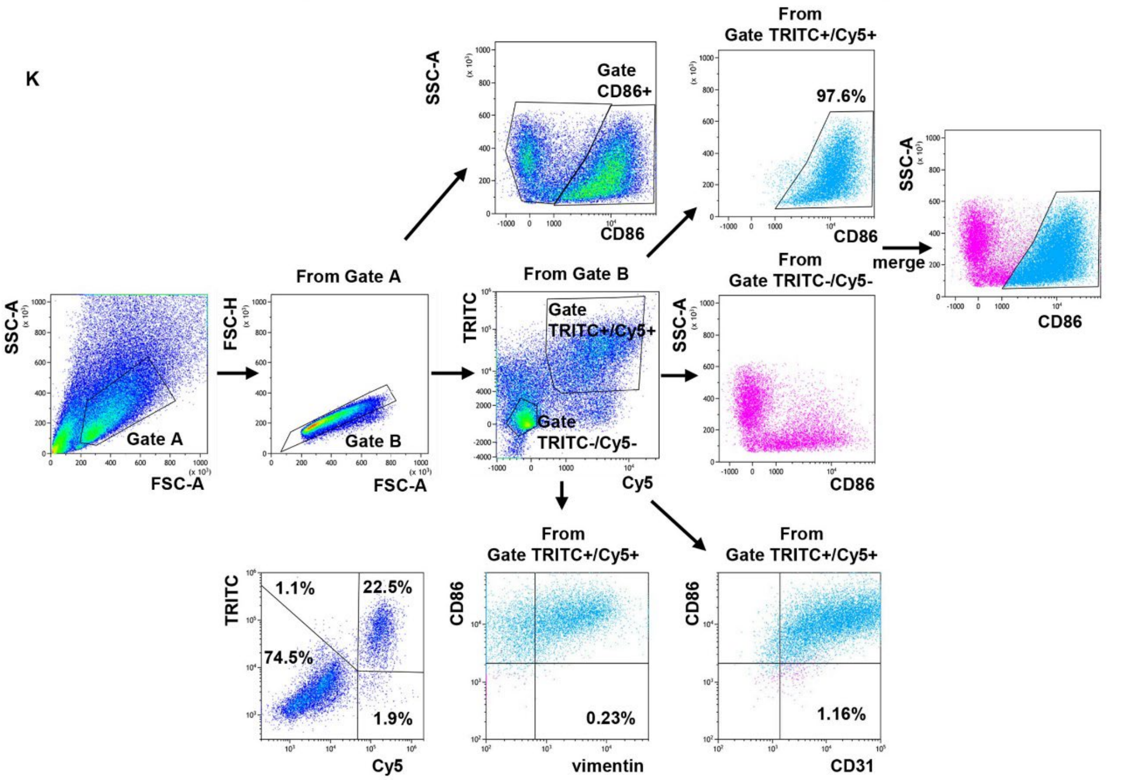

L

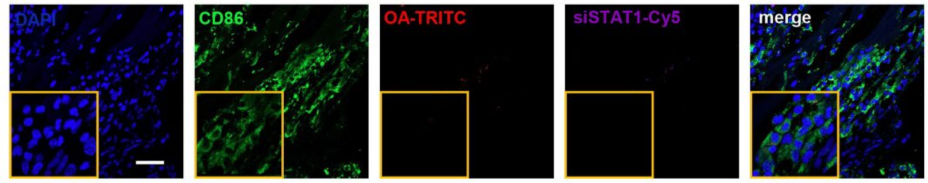

Fig. S3. (A) Flow cytometric analysis of folate receptor β (FRβ) expression in different cells. (B) Statistical analysis of the mean fluorescence intensity of FRβ expression in different cells as detected by flow cytometry, n = 3 per group. (C) Immunostaining of MI model mice on Day 3 post-MI and sham mice for CD86 (green) and FRβ (red), with DAPI counterstaining for nuclei (blue) in the ischemic heart injury border zones. (D) Gating strategy for flow cytometry to compare the proportion of FRβ-positive cells with various cell types in the infarcted area. (E) Clustering plot of single-cell sequencing results for Cy5-positive and Cy5-negative cells. (F) Feature plot of CD86-positive cells in for Cy5-positive and Cy5-negative cells. (G) Proportions of CD86-positive cells in two groups (73.3% in Cy5-positive cells and 9% in Cy5-negetive cells). (H) Summary of cell components contained in Cy5-positive cells. (I) and (J) Infrared spectra of OA-NO2 before and after TRITC modification. The red box highlights the amide bond formed by TRITC modification. The peaks in the 1650-1750 cm^{-1} range are characteristic of amide bonds (K) Gating strategy for flow cytometry testing the TRITC-positive, Cy5-positive and CD86-positive cells. (L) Immunofluorescence detection of the proportion of CD86-positive cells in the infarcted area phagocytosing TRITC-/Cy5- nanoparticles. scale bar = 50 μm. OA: nitro-oleic acid. MI: Myocardial infarction. FA: Folate Acid. TRITC: Tetramethylrhodamine isothiocyanate. Data are presented as means ± SD, $^{*}P<0.05$, $^{**}P<0.01$, $^{***}P<0.001$.

Fig. S4

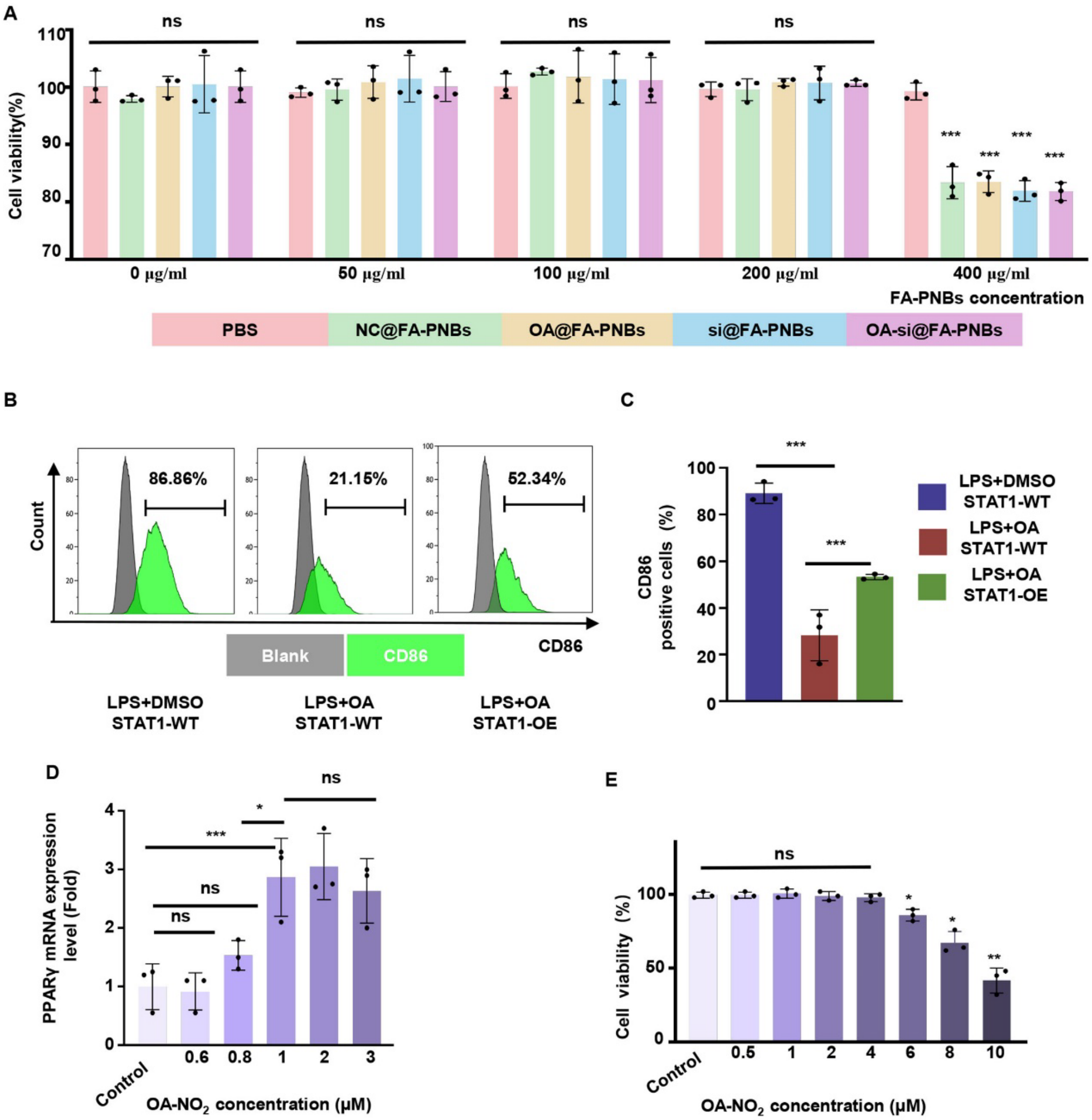

Fig. S4. (A) CCK8 assay to determine the safe concentration of nanodrugs for RAW 264.7 macrophages.Pink: control group treated with PBS. Green: macrophages treated with NC@FA-PNBs. Yellow: macrophages treated with OA@FA-PNBs. Blue: macrophages treated with si@FA-PNBs. Purple: macrophages treated with OA-si@FA-PNBs. ***Compared with PBS group. (B-C) Flow cytometry was used to detect the proportion of CD86-positive cells among LPS-induced WT macrophages or STAT1-OE macrophages regulated by OA-NO_2, and to compare the differences between these three groups. (D) qRT–PCR quantification of PPAR-γ activated by different concentrations of OA-NO_2. (E) CCK8 assay to determine the safe concentration of OA-NO_2 for macrophages. WT: wild-type. OE: Over Expression. OA: nitro-oleic acid. *and ** Compared with control group. The data are expressed as the means ± SD, n = 3 per group. *P<0.05, **P<0.01, ***P<0.001, ns: no significance.

Fig. S5

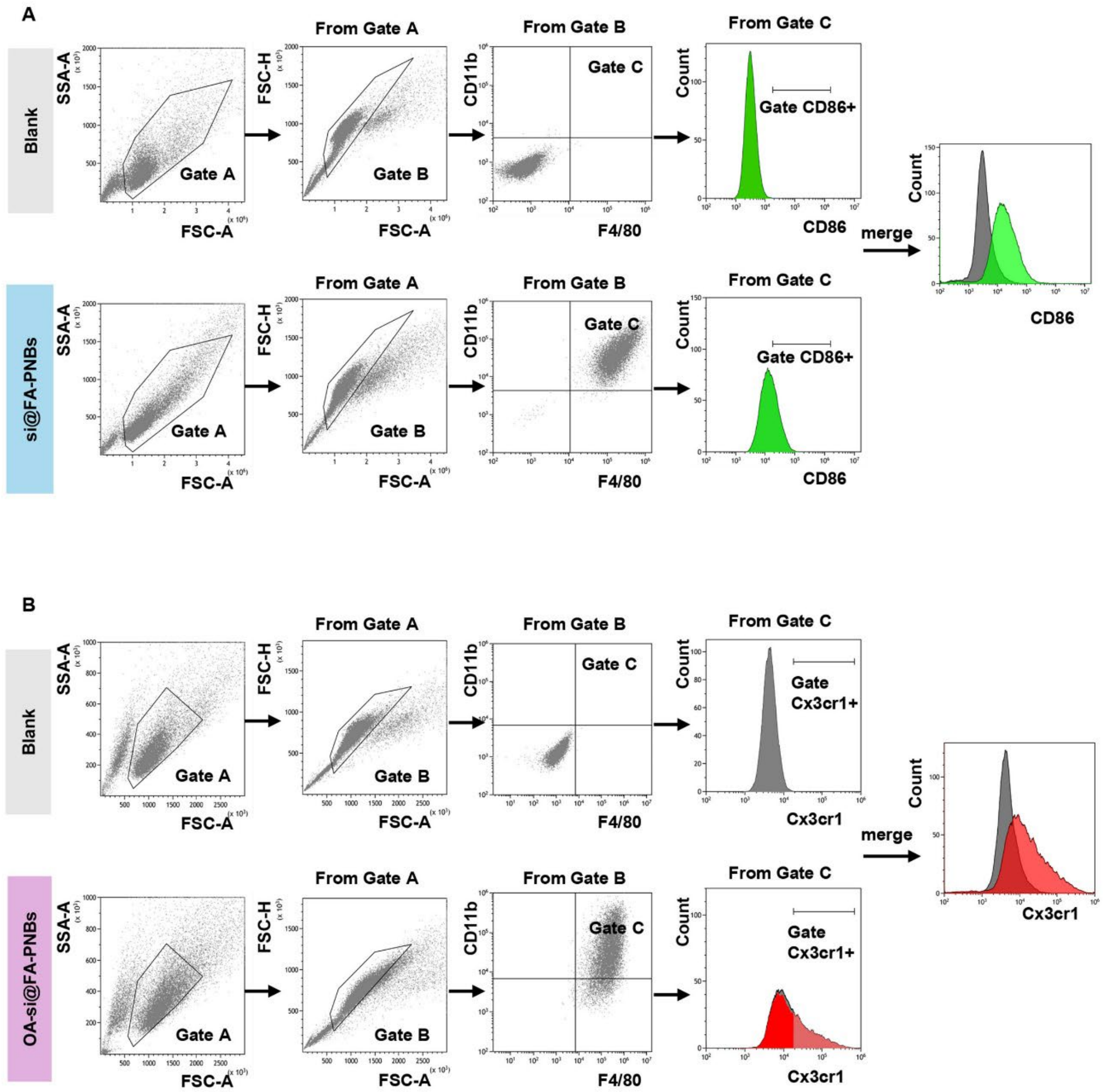

Fig. S5. The gating strategies of flow cytometry to determine the CD86-positive (A) or Cx3cr1-positive cells (B).

Fig. S6

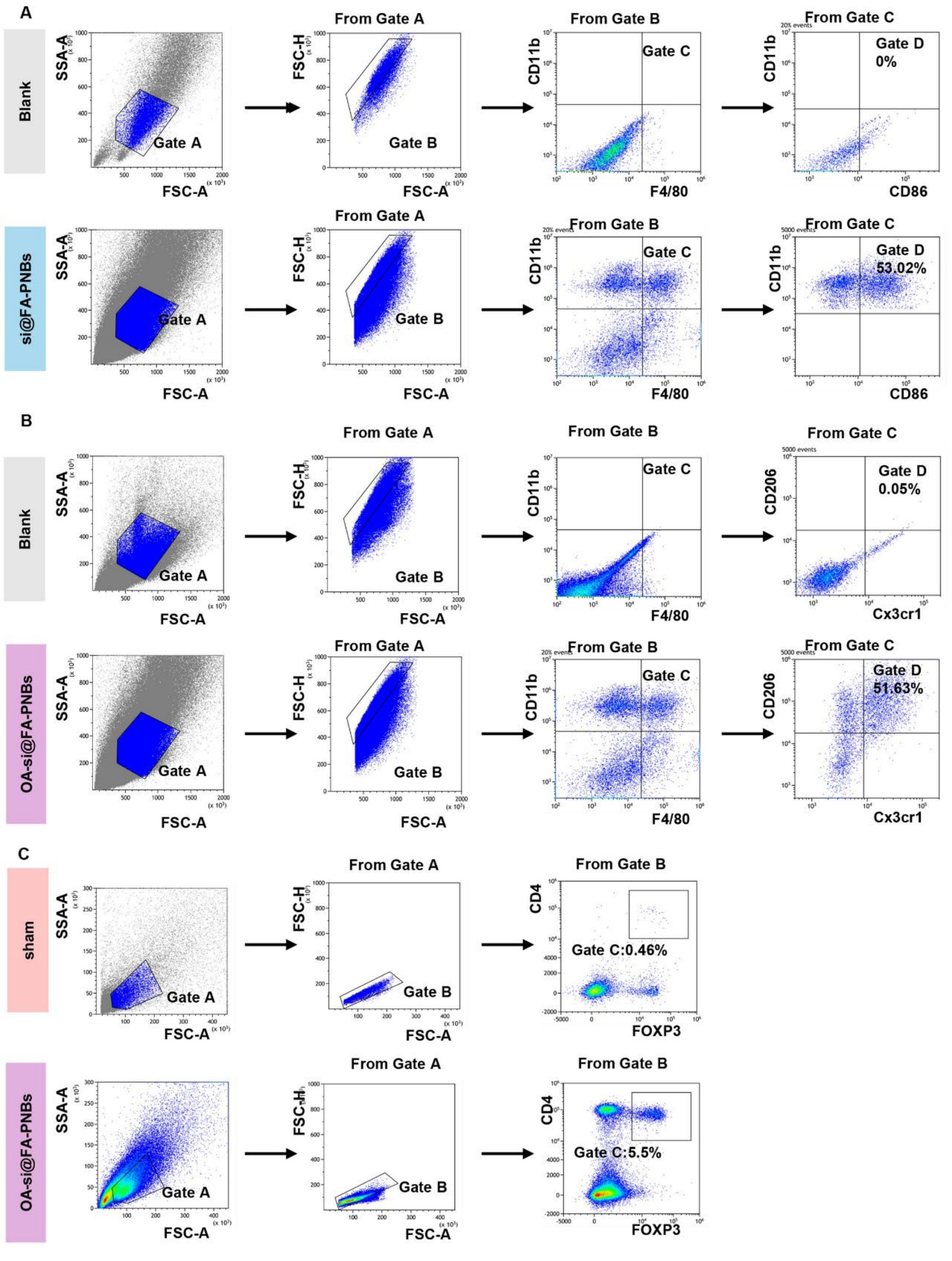
A
Blank
si@FA-PNBs
SSA-A
FSC-A
FSC-H
Gate A
Gate B
From Gate A
From Gate B
From Gate C
CD11b
F4/80
Gate C
CD86
Gate D
0%
53.02%
B
Blank
OA-si@FA-PNBs
CD206
Cx3cr1
Gate D
0.05%
51.63%
C
sham
OA-si@FA-PNBs
CD4
FOXP3
Gate C:0.46%
Gate C:5.5%

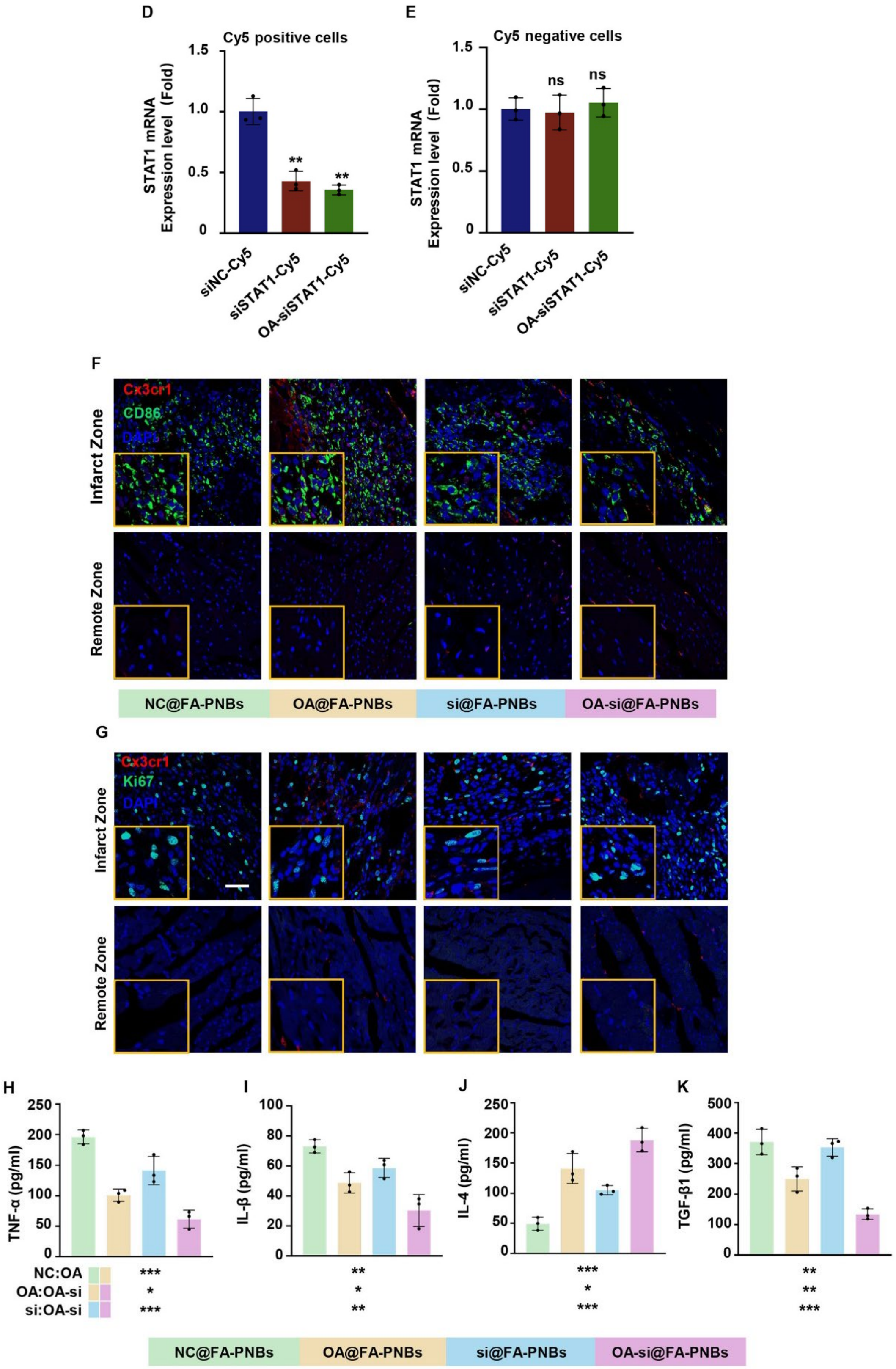

D
Cy5 positive cells
STAT1 mRNA Expression level (Fold)
1.5
1.0
0.5
0
**
**
siNC-Cy5
siSTAT1-Cy5
OA-siSTAT1-Cy5
E
Cy5 negative cells
STAT1 mRNA Expression level (Fold)
1.5
1.0
0.5
0
ns
ns
siNC-Cy5
siSTAT1-Cy5
OA-siSTAT1-Cy5
F
Cx3cr1
CD86
DAPI
Infarct Zone
Remote Zone
NC@FA-PNBs
OA@FA-PNBs
si@FA-PNBs
OA-si@FA-PNBs
G
Cx3cr1
Ki67
DAPI
Infarct Zone
Remote Zone
H
TNF-α (pg/ml)
250
200
150
100
50
0
I
IL-β (pg/ml)
100
80
60
40
20
0
J
IL-4 (pg/ml)
250
200
150
100
50
0
K
TGF-β1 (pg/ml)
500
400
300
200
100
0
NC:OA
OA:OA-si
si:OA-si
*** ** *** **
* * * **
*** ** *** ***
NC@FA-PNBs
OA@FA-PNBs
si@FA-PNBs
OA-si@FA-PNBs

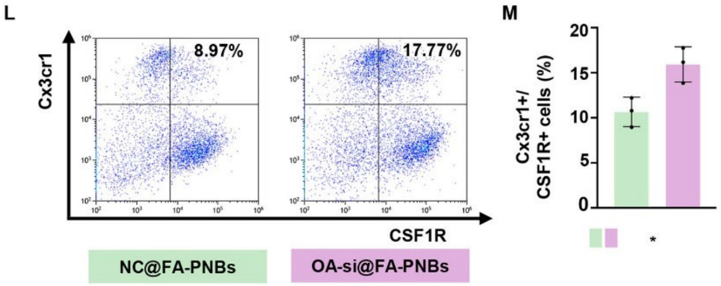

Fig. S6. We administered the corresponding intervention three days after establishing the MI mouse model, and subsequently, on the seventh day post-MI, we collected samples for experiments such as immunofluorescence and flow cytometry. (A-C) Gating strategy for flow cytometry analysis of CD86-positive macrophages, Cx3cr1-positive macrophages, and Tregs in the infarcted region of MI mice in different treatment groups. (D) qPCR analysis of STAT1 in Cy5-positive cells sorted from the hearts of MI mice 7 days post-MI. siNC-Cy5 indicates MI mice injected with Cy5-labeled NC-empty siRNA@FA-PNBs via tail vein. siSTAT1-Cy5 indicates MI mice injected with Cy5-labeled siSTAT1@FA-PNBs via tail vein. OA-siSTAT1-Cy5 indicates MI mice injected with Cy5-labeled OA-siSTAT1@FA-PNBs via tail vein. ** Compared with siNC-Cy5 group. (E) qPCR analysis of STAT1 in Cy5-negative cells sorted from the hearts of MI mice 7 days post-MI. ns: compared with siNC-Cy5 group. (F) Immunostaining of MI model mice on Day 7 post-MI for the Cx3cr1 (red) and CD86 (green), with DAPI counterstaining for nuclei (blue) in the ischemic heart injury border zones. scale bar = 50 μm. (G) Immunostaining of MI model mice on Day 7 post-MI for the Cx3cr1 (red) and Ki67 (green), with DAPI counterstaining for nuclei (blue) in the ischemic heart injury remote and infarct zones. scale bar = 50 μm. (H-K) Concentrations of TNF-α, IL-1β, IL-4 and TGF-β1 cytokines in myocardial tissue 7 days post-MI. (L) Flow cytometry analysis and (M) its results of Cx3cr1 and CSF1R double-positive cells 7 days post-MI. CSF1R: Colony-Stimulating Factor 1 Receptor. The data are expressed as means ± SD, n = 3 per group. *P<0.05, **P<0.01, ***P<0.001, ns: no significance.

Fig. S7

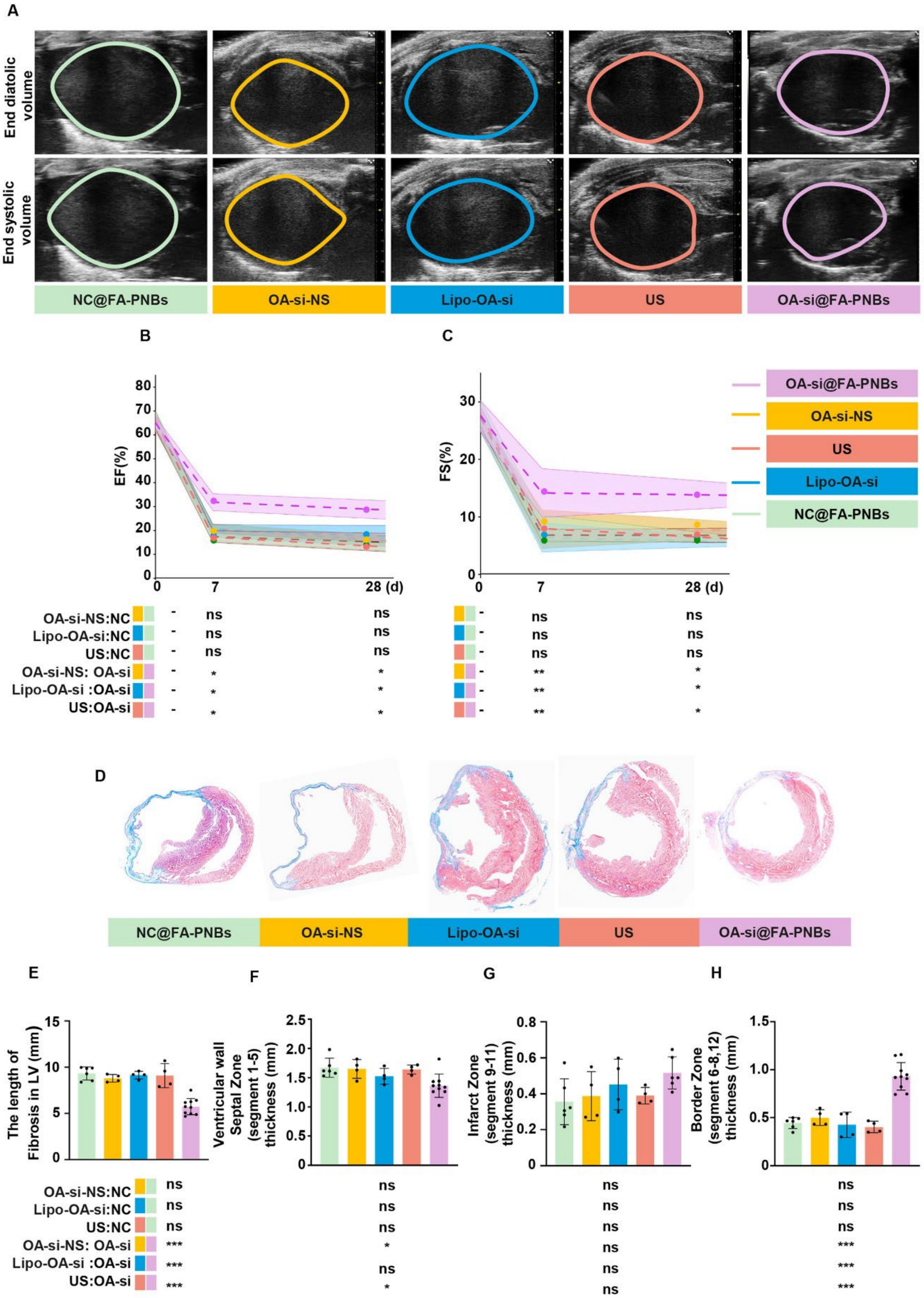

A
End diatolic volume
End systolic volume
NC@FA-PNBs
OA-si-NS
Lipo-OA-si
US
OA-si@FA-PNBs
B
EF(%)
28 (d)
C
FS(%)
OA-si-NS:NC
Lipo-OA-si:NC
US:NC
OA-si-NS: OA-si
Lipo-OA-si :OA-si
US:OA-si
D
E
The length of Fibrosis in LV (mm)
F
Ventricular wall Septal Zone (segment 1-5) thickness (mm)
G
Infarct Zone (segment 9-11) thickness (mm)
H
Border Zone (segment 6-8,12) thickness (mm)

Fig. S7. (A) Echocardiography of LV long-axis end diastolic/systolic volume in the three treatment groups 28 days post-MI compared with the NC@FA-PNBs. (B, C) Assessment of LV long-axis EF and FS before MI in the treatment and MI-only groups at 7 and 28 days post-MI, n=6 per group. (D) Heart Masson staining of treatment and MI-only groups at day 28 post-MI: fibrosis area (blue) and myocytes (red). (E) Quantitative analysis of infarct zone fibrosis length, and (F-H) Quantitative analysis of LV wall thickness in the septal zone, infarct zone, and border wall. n=4 per group. NC@FA-PNBs indicates MI mice injected without siSTAT1 and OA-NO_2, OA-si-NS indicates MI mice injected with free OA-NO_2 and siSTAT1 via tail vein. Lipo-OA-si indicated MI mice injected with Lipo 3000-encapsulated siRNA and OA-NO_2 via tail vein. US group indicates MI mice subjected to precordial US irradiation without additional tail vein injection treatments. The data are expressed as means ± SD. OA: nitro-oleic acid. ns: no significance.

Fig. S8

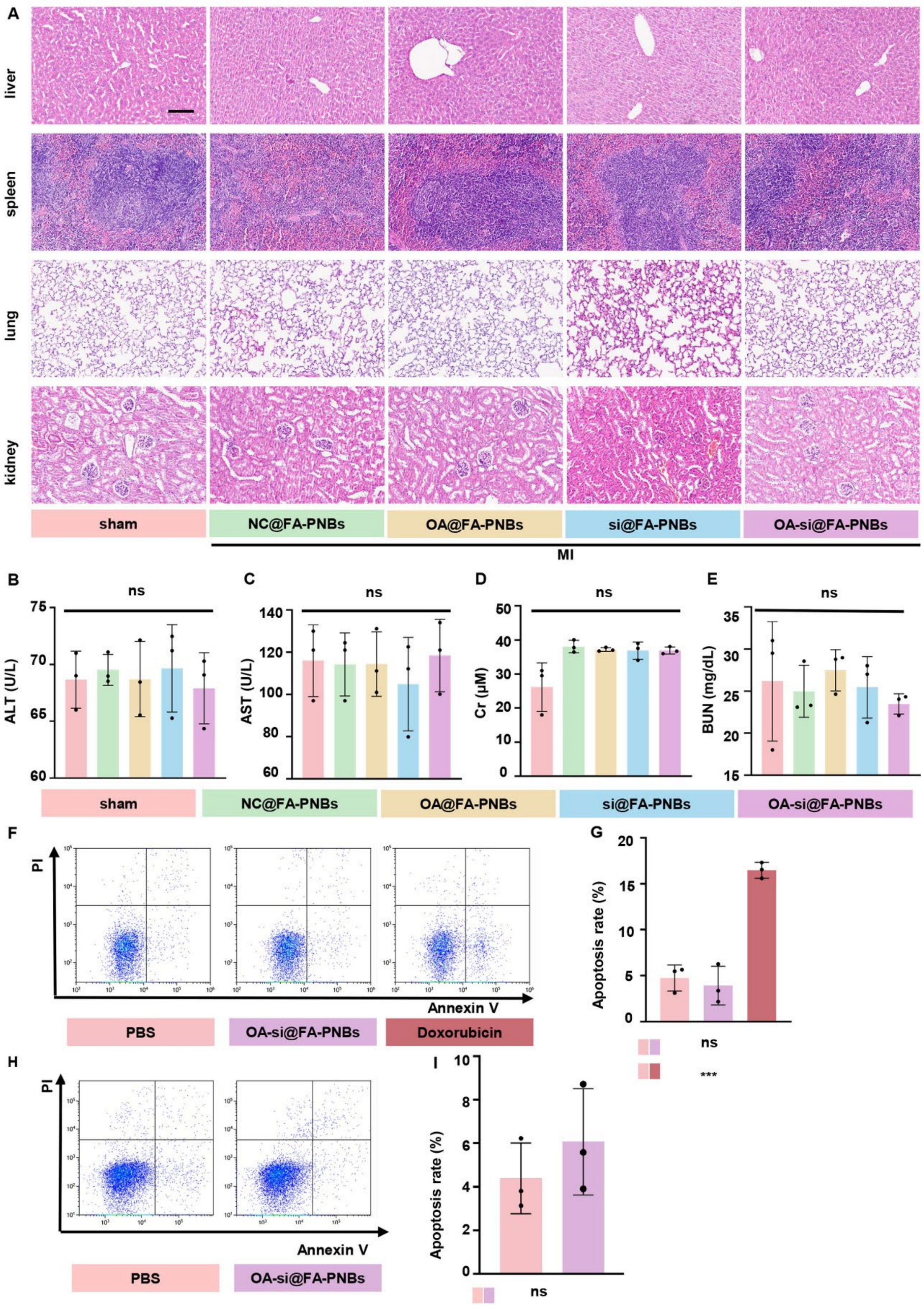
A
liver
spleen
lung
kidney
sham
NC@FA-PNBs
OA@FA-PNBs
si@FA-PNBs
OA-si@FA-PNBs
MI
B
C
D
E
ns
ALT (U/L)
AST (U/L)
Cr (μM)
BUN (mg/dL)
F
G
PI
Annexin V
PBS
OA-si@FA-PNBs
Doxorubicin
Apoptosis rate (%)
***
H
I

Fig. S8. (A) H&E images of main organs (liver, spleen, lung, and kidney) harvested from mice 28 days after different treatments, scale bar = 100 μm. (B-E) Serum levels of alanine aminotransferase (ALT), aspartate aminotransferase (AST), creatinine (Cr), and blood urea nitrogen (BUN) in mice that received each indicated treatment for 28 days. The reference ranges for ALT, AST, Cr, and BUN were 10.06-96.47U/L, 36.31-235.48U/L, 10.91-85.09 μM, and 10.81-34.74 mg/dL. (F) PBS, FA-PNBs, and doxorubicin were locally administered into the myocardium of normal mice. Two days post-injection, hearts were harvested and processed into single-cell suspensions. Flow cytometry was performed using the Annexin V-PI assay to assess apoptotic cell populations. (G) specifically quantifying the proportion of Annexin V-positive cells across different treatment groups. (H) Flow cytometry analysis using Annexin V and PI to assess the effects of FA-PNBs on apoptosis in HL-1 cells. PBS and FA-PNBs were added to the supernatant of HL-1 cell cultures. After 48 hours, the proportion of Annexin V-positive cells, representing apoptotic cell populations, was assessed in both groups. (I). Control group was treated with PBS. The data are expressed as means ± SD, n = 3 per group. ***P<0.001, ns: no significance.

Table S1. Sequences of STAT1 siRNAs.

ON-TARGET gene	Forward (5'~3')	Reverse (5'~3')
STAT1	GCCGAGAACAUACCAGAGAAUTT	AUUCUCUGGUAUGUUCUCGGCTT

Table S2. Primers used in this study.

gene	Forward	Reverse
STAT1	5’-TTTGTTCCCTTTCAGACCACCT-3’	5’-TCAGGAAGAAGGAGAGATTCCTGG -3’
Ki67	5’-ATCATTGACCGCTCCTTTAGGT -3’	5’-GCTCGCCTTGATGGTTCCT -3’
CSF1	5’-ATGAGCAGGAGTATTGCCAAGG -3’	5’-TCCATTCCCAATCATGTGGCTA-3’
CD86	5’-ATGGACCCCAGATGCACCAT -3’	5’-CCAGCTCACTCAGGCTTATGT -3’
TNF-α	5’-AGGACACCATGAGCACTGAAAGC -3’	5’-AAGGAGAAGAGGCTGAGGAACAAG -3’
IL-1β	5’-ATGCCACCTTTTGACAGTGATG-3’	5’-TGATGTGCTGCTGCGAGATT -3’
NF-κB	5’-GGCTACAACTCTGCAAACTGC-3’	5’-TCCCGGAGTTCATCTCATAGT-3’
Cx3cr1	5’-GAGTATGACGATTCTGCTGAGG-3’	5’-CAGACCGAACGTGAAGACGAG -3’
IL-4	5’-GAATAGGCCGGTCCAATCAGA -3’	5’-CAGCCATTCGTCGGACACATT -3’
PPAR-γ	5’-ATTCTCAGTGGAGACCGCC -3’	5’-AGGAACACGTTGTCAGCGG -3’
TGF-β1	5’-ACGTGGAAATCAACGGGATCAG -3’	5’-TAGTTGGTATCCAGGGCTCTC -3’
β-actin	5’-AGGTCGGTGTGAACGGATTTG -3’	5’-TGTAGACCATGTAGTTGAGGTCA-3’

Table S3. Primary antibody.

	Conjugate	Dilution	Incubation time	Incubation temperature	Host species
CD11b	APC	10^6 cells/ 100 µL	20 minutes	room temperature	Mouse
F4/80	PE	10^6 cells/ 100 µL	20 minutes	room temperature	Mouse
CD86	FITC	10^6 cells/ 100 µL	20 minutes	room temperature	Mouse
CD86	BV605	10^6 cells/ 100 µL	20 minutes	room temperature	Mouse
Cx3cr1	FITC	10^6 cells/ 100 µL	20 minutes	room temperature	Mouse
CD4	FITC	10^6 cells/ 100 µL	20 minutes	room temperature	Mouse
Foxp3	PE	10^6 cells/ 100 µL	20 minutes	room temperature	Mouse
FRβ	PE	10^6 cells/ 100 µL	20 minutes	room temperature	Mouse
CD31	Cy7	10^6 cells/ 100 µL	20 minutes	room temperature	Mouse
Vimentin	FITC	10^6 cells/ 100 µL	20 minutes	room temperature	Mouse
CD11b	Unconjugated	1:400	16 hours	4 Celsius	Rabbit
CD86	Unconjugated	1:400	16 hours	4 Celsius	Rat
Cx3cr1	Unconjugated	1:100	16 hours	4 Celsius	Rabbit
Ki67	Unconjugated	1:100	16 hours	4 Celsius	Rat
CSF1R	Unconjugated	1:100	16 hours	4 Celsius	Rabbit
